# Healthcare fragmentation, multimorbidity, potentially inappropriate medication, and mortality: a Danish nationwide cohort study

**DOI:** 10.1186/s12916-023-03021-3

**Published:** 2023-08-15

**Authors:** Anders Prior, Claus Høstrup Vestergaard, Peter Vedsted, Susan M. Smith, Line Flytkjær Virgilsen, Linda Aagaard Rasmussen, Morten Fenger-Grøn

**Affiliations:** 1 Research Unit for General Practice, Bartholins Allé 2, 8000 Aarhus C, Denmark; 2 Department of Public Health, Aarhus University, Aarhus C, Denmark; 3 Department of Clinical Medicine, Aarhus University, Aarhus C, Denmark; 4 Discipline of Public Health and Primary Care, Trinity College, University of Dublin, Dublin, Ireland

**Keywords:** Fragmentation, Continuity of care, Healthcare utilization, Multimorbidity, Primary care

## Abstract

**Background:**

Patients with multimorbidity are frequent users of healthcare, but fragmented care may lead to suboptimal treatment. Yet, this has never been examined across healthcare sectors on a national scale. We aimed to quantify care fragmentation using various measures and to analyze the associations with patient outcomes.

**Methods:**

We conducted a register-based nationwide cohort study with 4.7 million Danish adult citizens. All healthcare contacts to primary care and hospitals during 2018 were recorded. Clinical fragmentation indicators included number of healthcare contacts, involved providers, provider transitions, and hospital trajectories. Formal fragmentation indices assessed care concentration, dispersion, and contact sequence. The patient outcomes were potentially inappropriate medication and all-cause mortality adjusted for demographics, socioeconomic factors, and morbidity level.

**Results:**

The number of involved healthcare providers, provider transitions, and hospital trajectories rose with increasing morbidity levels. Patients with 3 versus 6 conditions had a mean of 4.0 versus 6.9 involved providers and 6.6 versus 13.7 provider transitions. The proportion of contacts to the patient’s own general practice remained stable across morbidity levels. High levels of care fragmentation were associated with higher rates of potentially inappropriate medication and increased mortality on all fragmentation measures after adjusting for demographic characteristics, socioeconomic factors, and morbidity. The strongest associations with potentially inappropriate medication and mortality were found for ≥ 20 contacts versus none (incidence rate ratio 2.83, 95% CI 2.77–2.90) and ≥ 20 hospital trajectories versus none (hazard ratio 10.8, 95% CI 9.48–12.4), respectively. Having less than 25% of contacts with your usual provider was associated with an incidence rate ratio of potentially inappropriate medication of 1.49 (95% CI 1.40–1.58) and a mortality hazard ratio of 2.59 (95% CI 2.36–2.84) compared with full continuity. For the associations between fragmentation measures and patient outcomes, there were no clear interactions with number of conditions.

**Conclusions:**

Several clinical indicators of care fragmentation were associated with morbidity level. Care fragmentation was associated with higher rates of potentially inappropriate medication and increased mortality even when adjusting for the most important confounders. Frequent contact to the usual provider, fewer transitions, and better coordination were associated with better patient outcomes regardless of morbidity level.

**Supplementary Information:**

The online version contains supplementary material available at 10.1186/s12916-023-03021-3.

## Background

Patients with multiple long-term conditions, i.e., multimorbidity, are frequent users of services in all healthcare sectors [1–3]. Despite increased healthcare delivery, they report impaired daily functioning, poor quality of life, and adverse health outcomes [4–7]. For these patients, the coordination of care is often complicated by the high number of clinicians involved in their treatment, including multiple appointments, involvement of both primary care and specialists in secondary care, repeated referrals, and parallel outpatient trajectories with duplicate services in a highly specialized healthcare system [1, 8]. This may lead to inadequate transfer of information, unclear treatment responsibilities, and ultimately fragmented healthcare. Care fragmentation produces adverse consequences, including economic inefficiency, inequality in health, and depersonalization of the patient [9]. Furthermore, poor continuity of care has also been linked to more hospital admissions, inappropriate medication use, and increased mortality [10–17].

The coordinating role of the general practitioner (GP) is a cornerstone in the universal healthcare system in Denmark. However, the extensive care required for treating patients with multiple long-term conditions is regarded as challenging, fragmented, and uncoordinated, and the GPs report to have little time and limited capacity [18–22]. The patients with multimorbidity report a lack of holistic patient-centered care and high levels of treatment burden, i.e., the work required by patients to manage their conditions [23–25]. By definition, integrated care aims to incorporate service delivery designed to create connectivity, alignment, and collaboration within the care sectors [26], but it has been challenged by changes in healthcare provision, e.g., strong specialization (so-called silo structures) in hospital care, new requirements from guidelines, and an aging population with complex needs in a healthcare system with limited resources [2, 8, 27–29].

Different measures of the spectrum between continuity of care and care fragmentation have been developed to describe the distribution of care among providers, concentration on a single health provider, or transitions between providers [30]. Yet, previous studies on care fragmentation and its consequences have not considered the healthcare system on a national scale. The comprehensive Danish health registers provide a unique opportunity to study care fragmentation across all healthcare sectors at a population level.

We aimed to quantify care fragmentation using various clinical indicators and formal indices and to analyze the associations with potentially inappropriate medication and all-cause mortality in consideration of the level of patient morbidity.

## Methods

### Design and study population

We performed a nationwide, register-based cohort study. The study population included all Danish citizens aged ≥ 18 years on 1 January 2018. Data on healthcare contacts was obtained from 1 January 2017 until 31 December 2017, and the cohort was then followed from 1 January 2018 until death, emigration, or end of study (31 December 2018), whichever came first.

### Setting

The Danish universal healthcare system is mainly publicly funded, and residents have free access to medical services by GPs, private practice specialists, and the hospital system. A total of 99% of Danish citizens are listed with a general practice, which provides the first point of contact for medical advice [31]. Each practice comprises approximately 1600 listed patients per full-time GP. Around half of all clinics are single-handed. The GP acts as a gatekeeper, and referrals are needed to the hospital and most specialists, except for otolaryngologists and eye specialists [32].

GPs work as independent primary care contractors for the health authorities and are remunerated through a mix of per capita and fee-for-service payments [33]. Remunerated services include daytime and out-of-hours consultations and specific chronic care services [3]. Public hospitals provide emergency services, outpatient ambulatory services, and inpatient services. Some services are contracted with private hospitals.

### Data sources

This study was based on data from the Danish national health registries. The data was linked at the individual level through the unique 10-digit personal identification code assigned to all Danish citizens at birth or immigration [31, 34]. The Danish national registers hold complete high-quality and validated data at the individual level on age, sex, civil and vital status [34], population density, household income, educational attainment [31], redeemed medicine prescriptions [35], date and type of primary care and out-of-hours contacts [36], date of outpatient contacts and hospital admissions, discharge ICD-10 diagnoses, and procedures at public and private hospitals [37]. To assess disease and multimorbidity status for patients, we utilized the Danish Multimorbidity Index algorithm, which provides information on 39 long-term physical and mental conditions (see Additional file 1: Methods S1 for definitions) [4].

### Measures of care fragmentation

To assess healthcare utilization and care trajectories, we constructed a dataset containing all contacts to healthcare providers in the primary healthcare sector and the hospital sector at the provider level, i.e., primary clinics or hospital departments. Primary care providers included GP clinics (daytime and out-of-hours services) and publicly funded private practice specialists. Primary care contacts were identified through the unique clinic number. We had no data on which physician the patient saw in the clinic [36]. Using the Patient List Register, we obtained the number of contacts to own GP clinic versus other providers. Hospital contacts included all contacts to inpatient clinics (hospital admissions), outpatient clinics, and emergency rooms. Hospital providers included public and private hospitals departments, where the place of contact was identified by combining hospital identification number, department codes, and specialty codes [37]. A hospital trajectory was defined as the period between the first and the last visit to a hospital outpatient clinic or from hospital admission to discharge (see Additional file 1: Methods S2 for a visualized example of a patient pathway).

Several clinical indicators of care fragmentation were included: total number of contacts to healthcare providers, number of different providers involved (clinics and departments), number of different GP clinics involved (daytime and out-of-hours services), number of transitions between different providers, and number of hospital trajectories for each patient over the study period (Table 1). Moreover, we assessed the number of transitions between hospital trajectories and the number of overlaps between parallel hospital trajectories, i.e., periods of time when the patient visited several outpatient clinics or had overlapping hospital admissions.

**Table 1:** Measures of cross-sectoral care fragmentation

Category	Measure	Description
*Clinical indicators of care fragmentation*	Total contacts	Overall number of contacts to all healthcare providers*
	Involved providers	Number of different providers involved
	Involved GP clinics	Number of different GP clinics involved (daytime or out-of-hours)
	Provider transitions	Number of transitions between different providers, i.e., not seeing the same provider they saw at their last contact
	Hospital trajectories	Overall number of outpatient ambulatory care trajectories and hospital admissions
	Hospital trajectory transitions	Number of initiated/closed ambulatory care trajectories or hospital admissions/discharges
	Hospital trajectory overlaps	Number of overlapping hospital trajectories
Formel fragmentation indices**	Usual Provider of Care Index (UPC)***	Concentration of care with a single provider; the proportion of contacts to the provider whom the patient visited most times
	Continuity Of Care Index (COCI)***	Distribution of care among providers, weighting both the frequency and dispersion of contacts
	Sequential Continuity Index (SECON)***	Degree of transitions based on number of contacts to the provider whom the patient visited most recently
	Known GP Index***	Proportion of contacts to the patient’s own GP clinic out of all provider contacts

For a patient pathway example, see Additional file 1: Methods S2, details in Pollack et al. 2016 [30]

^*^Providers: GP clinics (daytime and out-of-hours services), publicly funded private practice specialists (e.g., otolaryngologists, ophthalmologists, or dermatologists), or hospital departments (emergency room, inpatient and outpatient services)

^**^All formal care fragmentation indices range from 0 to 1, with lower values indicating a higher degree of care fragmentation

$$^{***}UPC=\underset{}{\mathrm{max}}\left(\frac{{n}_{i}}{n}\right) COCI=\frac{\left({\sum }_{i=1}^{p}{n}_{i}^{2}\right)-n}{n(n-1)} SECON=\frac{{\sum }_{j=1}^{n-1}{c}_{j}}{n-1} Known\,GP\,Index=\frac{{n}_{own}}{n}$$

*n*, total number of contacts; *n*_*i*_, number of contacts to provider *i*; *p*, total number of providers; *c*_*j*_, takes 1 if contacts *j* and *j* + *1* are to the same provider, otherwise 0; *n*_*own*_, number of contacts to the patient’s own GP clinic

Additionally, we included formal fragmentation indices, which provide a mathematical quantification of different aspects of fragmentation [30, 38–41]: (1) the Usual Provider of Care Index (UPC) which describes the concentration of contacts with a single provider, (2) the Bice-Boxerman Continuity of Care Index (COCI) which describes the distribution of care among providers, and (3) the Sequential Continuity Index (SECON) which describes the number of contacts to the provider whom the patient visited most recently (see Table 1 for details) [30, 42]. Additionally, we constructed the *Known GP Index* by calculating the proportion of contacts to the patient’s own GP clinic out of all healthcare contacts. All these indices ranged from 0 to 1, with lower values indicating a higher degree of care fragmentation. To ensure the robustness of the indices, at least four healthcare contacts were required to calculate the indices as recommended by Rosenberg et al. [43].

### Outcomes

We had two main outcomes. The first, potentially inappropriate medication (PIM), was chosen as a clinical indicator of quality of care as it assesses days with potentially suboptimal medication regimes and is associated with adverse health outcomes such as emergency hospital admission [44]. It was based on a modified version of the STOPP/START criteria [45], which are used clinically and in pharmacoepidemiologic research to identify potentially inappropriate drug-drug and drug-disease combinations, e.g., stop concomitant use of drugs with anticholinergic properties or prolonged benzodiazepine use (STOPP criteria), or combinations that would suggest medication initiation, e.g., start antiplatelet therapy in patients with a history of coronary disease (START criteria). These criteria were adapted for an adult population in a Danish register-based setting through an iterative consensus group process, which resulted in the selection of 29 STOPP criteria. The process is described in detail elsewhere [46]. During the same process, 10 START criteria were also selected (Additional file 1: Methods S3). The process resulted in an algorithm to identify the periods of time when an individual was subject to PIM by combining data on redeemed drug prescriptions and diagnoses from the Danish registers. Patients may have contributed with PIM time more than once if being subjected to multiple concurrent PIMs for up to a maximum of the 1-year study period. Time with PIM was assessed between 1 January 2018 and 31 December 2018.

The second outcome, all-cause mortality, was chosen as an overall indicator of patient prognosis. Death was assessed during follow-up as recorded in the Danish Civil Registration System between 1 January 2018 and 31 December 2018 [34].

### Statistical analyses

Clinical indicators of care fragmentation were categorized into groups by count. Formal fragmentation indices were divided into groups with 0.25 increments from 0 to 1. Care fragmentation measures were presented as means and group distribution by the number of comorbid conditions.

Negative binomial regression models were used to estimate incidence rate ratios (IRR) with 95% confidence intervals (CIs) of total PIM time (sum of days for each PIM criteria), accounting for time at risk. A first model was adjusted for age group and sex. A second model was further adjusted for cohabitation status, country of origin, educational attainment according to the UNESCO educational level, OECD-adjusted household income, population density (urban vs rural areas), and presence of each of the 39 conditions in the Danish Multimorbidity Index.

Cox regression models were used to estimate all-cause mortality hazard ratios (HR) with 95% CIs, with age as the underlying time axis. Two models were constructed, with similar adjustments as in the models for PIM. Absolute terms were obtained as cumulative incidence proportions (CIP).

To visualize the functional form of the formal fragmentation indices, a restricted cubic spline model, covering the full range of values, was added with five knots using Harrell’s default percentiles.

The analyses were stratified on disease count at baseline to assess interactions between disease burden and care fragmentation. A sensitivity analysis was performed to examine individual STOPP/START criteria items.

All analyses were performed with Stata 17. The reporting of this study followed the STROBE guidelines.

## Results

### Patient characteristics and descriptive statistics

Table 2 shows the baseline characteristics of all the included patients (*N* = 4,651,842) and the sub cohort of patients with at least four contacts during the study year (*N* = 3,160,195, 68% of total) for which the UPC, COCI, SECON, and Known GP Index were available.

**Table 2:** Baseline characteristics of study population

**Variables**	**Total** Number (column %)	**At least four healthcare contacts** Number (column %)
Total	4,651,842 (100.0)	3,160,195 (100.0)
*Demographics*		
Sex		
Male	2,295,510 (49.3)	1,316,484 (41.7)
Female	2,35,6332 (50.7)	1,843,711 (58.3)
Age group (years)		
18–29	976,719 (21.0)	551,873 (17.5)
30–39	671,244 (14.4)	404,904 (12.8)
40–49	766,654 (16.5)	469,505 (14.9)
50–59	790,250 (17.0)	534,448 (16.9)
60–69	662,241 (14.2)	505,644 (16.0)
70–79	530,647 (11.4)	458,035 (14.5)
80–89	209,894 (4.5)	194,278 (6.1)
≥ 90	44,193 (1.0)	41,508 (1.3)
Population density (thousands)		
< 1	866,063 (18.6)	574,100 (18.2)
1–10	973,793 (20.9)	692,233 (21.9)
10–100	1,227,477 (26.4)	867,630 (27.5)
> 100	1,503,808 (32.3)	984,551 (31.2)
Unknown	80,701 (1.7)	41,681 (1.3)
*Socioeconomics*		
Cohabitation		
Single	1,831,216 (39.4)	1,212,755 (38.4)
Married	2,130,810 (45.8)	1,505,044 (47.6)
Cohabitating	689,816 (14.8)	442,396 (14.0)
Origin		
Danish	4,040,678 (86.9)	2,807,452 (88.8)
Descendant	533,733 (11.5)	306,040 (9.7)
Immigrant	77,431 (1.7)	46,703 (1.5)
Years of education		
≤ 10	1,208,988 (26.0)	875,085 (27.7)
11–15	2,12,4689 (45.7)	1,443,455 (45.7)
≥ 16	1,139,886 (24.5)	755,614 (23.9)
Unknown	178,279 (3.8)	86,041 (2.7)
Quintile of income		
1 (lowest)	709,194 (15.2)	443,465 (14.0)
2	810,511 (17.4)	622,784 (19.7)
3	923,454 (19.9)	656,784 (20.8)
4	1,053,053 (22.6)	700,817 (22.2)
5 (highest)	1,117,288 (24.0)	723,033 (22.9)
Unknown	38,342 (0.8)	13,312 (0.4)
*Morbidity*		
Disease count		
0	2,428,321 (52.2)	1,221,788 (38.7)
1	911,017 (19.6)	712,135 (22.5)
2	509,114 (10.9)	452,630 (14.3)
3	332,085 (7.1)	312,293 (9.9)
4	210,925 (4.5)	204,226 (6.5)
5	125,762 (2.7)	123,493 (3.9)
≥ 6	134,618 (2.9)	133,630 (4.2)

**Fig. 1:**
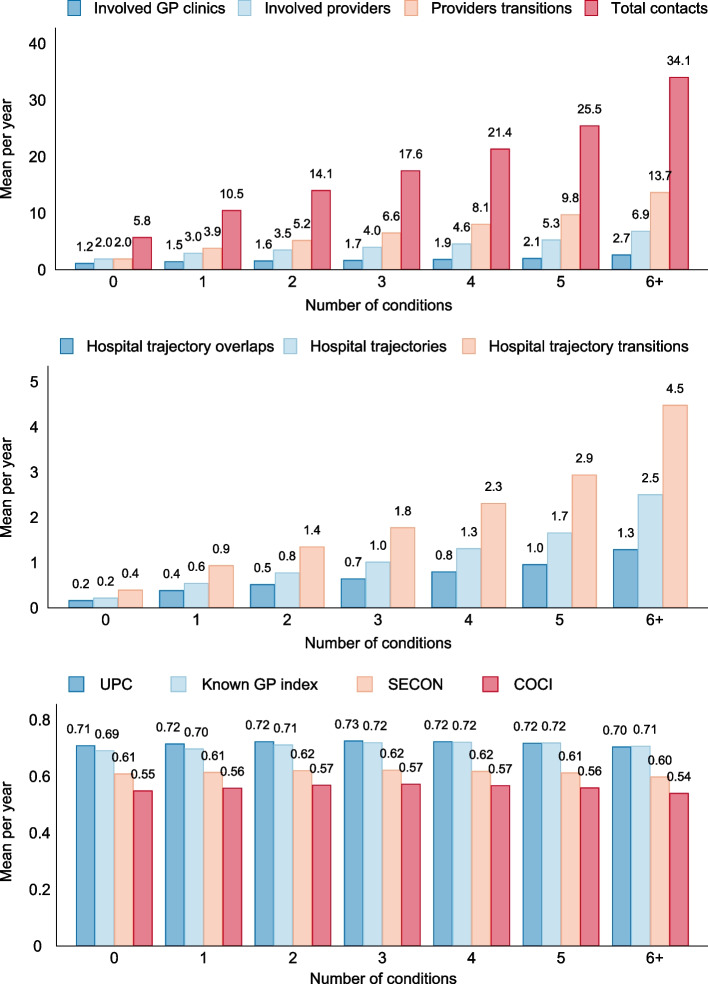
Mean of care fragmentation measures by number of conditions. UPC, Usual Provider of Care Index; SECON, Sequential Continuity Index; COCI, Continuity of Care Index; GP, general practitioner

Figure 1 shows the care fragmentation measures by morbidity level. The mean number of contacts increased from 5.8 in those with no long-term conditions to 34.1 in those with six or more long-term conditions of whom the majority had 20 or more contacts (Additional file 1: Figure S1). Those with three conditions had a mean of 4.0 involved providers (1.7 involved GP clinics) and 6.6 provider transitions, whereas those with six or more conditions had 6.9 providers (2.7 involved GP clinics) and 13.7 provider transitions. The level of morbidity was positively associated with the number of hospital trajectories, trajectory transitions, and overlaps. The proportion of hospital contacts ranged from 13% (no long-term conditions) to 17% (six or more conditions).

The mean values of the formal care fragmentation indices were largely independent on the number of conditions; the proportion of contacts with the patient’s own GP clinic ranged from 69% (0 conditions) to 72% (4–5 conditions) (Fig. 1). As the number of conditions increased, fewer patients experienced extremely high or extremely low levels of care fragmentation (Additional file 1: Figure S1).

### Clinical indicators of care fragmentation and patient outcomes

**Fig. 2:**
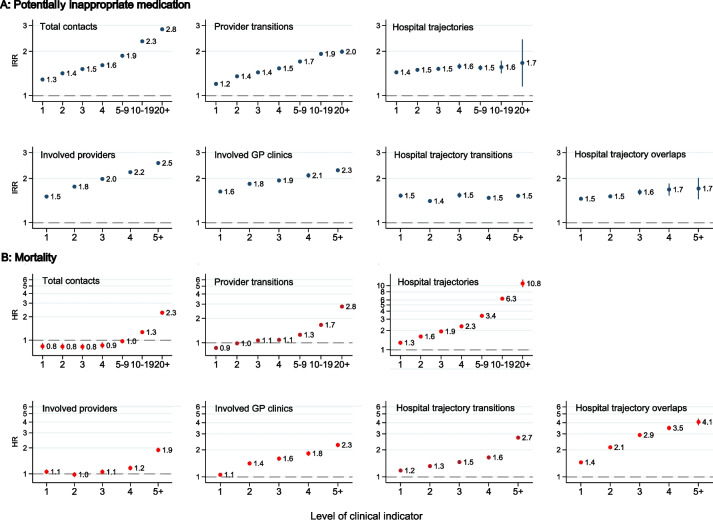
Potentially inappropriate medication and all-cause mortality by clinical indicators of fragmentation. Adjusted for age, sex, civil status, origin, educational attainment, income level, population density, and the 39 individual physical and mental conditions in the Danish Multimorbidity Index. Reference for clinical indicators = 0. IRR, incidence rate ratio; HR, hazard ratio

High levels of all clinical indicator of care fragmentation were associated with higher rates of PIM (Fig. 2, panel A), and higher all-cause mortality (Fig. 2, panel B), even after fully adjusting for demographic characteristics, socioeconomic factors, and the underlying combination of diseases. The strongest associations with PIM were found for 20 + contacts (IRR 2.83, 95% CI 2.77–2.90), 5 + involved providers (IRR 2.55, 95% CI 2.50–2.60), and 5 + involved GP clinics (IRR 2.28, 95% CI 2.21–2.35) compared with 0, respectively. The strongest associations with mortality were found for 20 + hospital trajectories (HR 10.8, 95% CI 9.48–12.4), 5 + hospital trajectory overlaps (HR 4.07, 95% CI 3.76–4.42), and 20 + provider transitions (HR 2.80, 95% CI 2.71–2.90) compared with 0, respectively (Additional file 1: Tables S1 and S2). Most indicators presented a dose–response relationship with both outcomes, but those with moderate contact levels (up to four contacts per year) and a single provider transition had slightly lower mortality.

### Formal fragmentation indices and patient outcomes

**Fig. 3:**
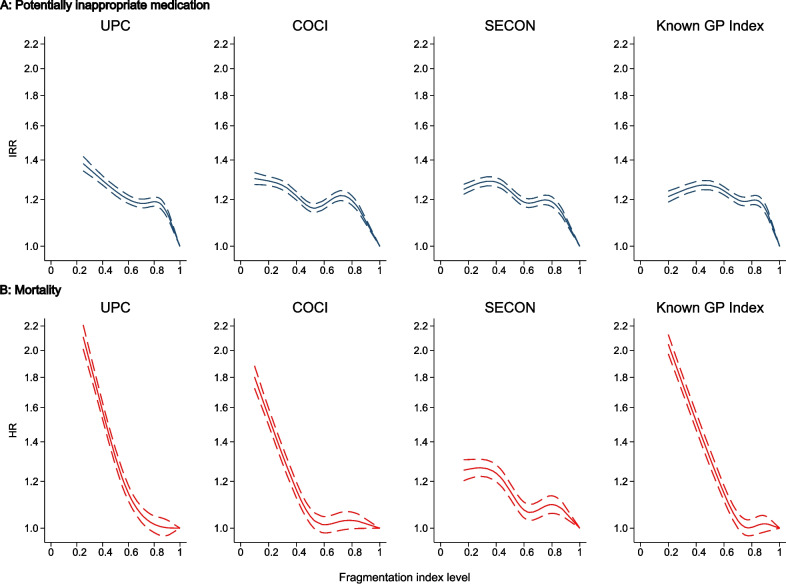
Potentially inappropriate medication and all-cause mortality by formal care fragmentation indices. Restricted cubic splines adjusted for age, sex, civil status, origin, educational attainment, income level, population density, and the 39 individual physical and mental conditions in the Danish Multimorbidity Index. Reference for formal fragmentation indices = 1. UPC, Usual Provider of Care Index; COCI, Continuity of Care Index; SECON, Sequential Continuity Index; GP, general practitioner; IRR, incidence rate ratio; HR, hazard ratio

High levels of formal fragmentation (as indicated by low values of the UPC, COCI, SECON, and the Known GP Index) were all associated with both potentially inappropriate medication (Fig. 3, panel A) and all-cause mortality (Fig. 3, panel B) after adjustments (Additional file 1: Tables S1 and S2). Having less than 25% of contacts with your usual provider was associated with more PIM and higher mortality (PIM IRR 1.49, 95% CI 1.40–1.58; mortality HR 2.59, 95% CI 2.36–2.84) compared with full continuity. Similar results were found with less than 25% of contacts to your own GP clinic (PIM IRR 1.24, 95% CI 1.21–1.28; mortality HR 2.48, 95% CI 2.36–2.60), for patients with the highest level of contact dispersion across providers (COCI) (PIM IRR 1.34, 95% CI 1.31–1.37; mortality HR 1.70, 95% 1.63–1.76), and for patients with lowest contact sequentially (SECON) (PIM IRR 1.30, 95% CI 1.27–1.34; mortality HR 1.39, 95% CI 1.33–1.46). A dose–response relationship was present for most associations.

### Stratified analyses

The stratified analyses on fragmentation measures showed the same overall pattern as the main analyses across different morbidity levels (Additional file 1: Figures S2 and S3). Some measures showed a negative interaction term with increasing morbidity for PIM, but a positive for mortality (involved GP clinics *p* for interaction all < 0.05; COCI *p* for interaction 0.800 to < 0.05). No consistent interaction pattern between fragmentation and the number of conditions was found.

The sensitivity analysis on the separate STOPP/START criteria for PIM showed that care fragmentation was associated with lack of deprescribing inappropriate medication and, to a lesser extent, not initiating appropriate medication (Additional file 1: Table S3).

## Discussion

### Summary of findings

This study showed that clinical indicators of care fragmentation, i.e., the number of contacts, involved providers, transitions, and hospital trajectories, increased with the number of chronic conditions. However, the level of formal fragmentation indices, including the proportion of own GP contacts, remained stable across morbidity level. High levels of fragmentation on all clinical indicators and formal fragmentation indices were associated with higher rates of PIM and increased mortality, even after adjusting for underlying conditions, demographics, and socioeconomic factors. A high number of contacts and providers, including GP clinics, showed the strongest association with PIM, whereas a high number of hospital trajectories, trajectory overlaps, and provider transitions showed the strongest association with mortality. Among the formal fragmentation indices, low values of UPC, i.e., few visits of all to the usual provider, had the strongest association with PIM and mortality. For the associations between fragmentation measures and patient outcomes, there were no consistent interactions with number of conditions.

### Interpretations

This study suggests that healthcare fragmentation could be an independent risk factor for adverse patient outcomes. Besides the number of contacts and providers, concentration of care on specific providers, dispersion of care across providers, and the sequence of transitions among providers all played a role in relation to patient outcomes. This indicates that many aspects of care fragmentation contribute to poorer prognosis.

The GP played a central role for most patients; 70% of all contacts was with the patient’s own GP, and only 13–17% of all contacts were with the hospital. For complex or progressive disease, it may be clinically appropriate that specialists and hospitals are involved; contact rates may be intensified, and more providers may be expected in the diagnostic process, treatment, and follow-up. However, to ensure coherence in care and enhance the patient experience of care, it is important that visits are coordinated and information is transferred in a timely way between healthcare professionals. The dose–response relationship between care fragmentation and adverse patient outcomes pointed to a systemic effect, which could indicate that optimal coordination and coherence in care is not being achieved in practice. Notably, primary care fragmentation, as measured by the number of involved GP clinics, was also associated with PIM and mortality.

Formal care fragmentation indices were found to be rather evenly distributed across morbidity levels in unadjusted models. For patients with multimorbidity, this could be explained by the proportionally higher number of contacts to the patient's own GP despite high numbers of contacts and involved providers. The associations between fragmentation measures and patient outcomes were consistent across morbidity levels; this may seem unexpected given the known association between PIM, multimorbidity, and polypharmacy. However, it may be explained by a higher number of contacts with GPs for patients with multimorbidity; they may maintain continuity of care through regular GP contact, thereby keeping their fragmentation indices low and mitigating potential adverse outcomes from care fragmentation.

### Comparison with existing literature

To our knowledge, this is the first to study to link nationwide cross-sectoral data on care fragmentation and patient outcomes. In previous studies, the mean level of the care fragmentation indices varied according to the population investigated and the methods used, but our estimates generally showed higher continuity of care levels than found in studies on primary care populations [41, 47]. Different continuity of care measures is correlated which was also the case in our study [30]. Our findings are in accordance with recent studies suggesting that care fragmentation is associated with more inappropriate medication [17] and increased mortality [10, 11]. This study examined longitudinal continuity of care based on administrative data. Informational and relational continuity of care describe other aspects of continuity of care, and the patient experience of continuity is often linked to the patient-professional relationship, i.e., seeing the same person to obtain interpersonal knowledge and trust [39, 48]. Having repeated coordinated contacts to the same provider is not necessarily the same as experiencing relational continuity, but it is a prerequisite [49]. Our analyses on provider level probably underestimates the association between relational continuity and patient outcomes.

### Strengths and limitations

The nationwide cohort design and the prospectively collected data from validated databases in all care sectors were major strengths of this study, which reduced selection bias and loss to follow-up [31]. Owing to the Danish registers, individual-level data were available for demographic, socioeconomic, and health variables. The concept of PIM reflected quality of care based on clinical practice guidelines and has been extensively validated internationally. The register-adapted definition of PIM provided an opportunity to assess a quality indicator on a national scale.

The study also had certain limitations. Administrative data were used to track contacts in clinics and departments, but we could not track which physician the patient had seen and the reason for encounter. Some variables (e.g., multimorbidity conditions and PIM categories) were aggregated from different data sources by algorithms that may have overestimated or underestimated conditions.

The association between provider contacts and health outcomes is susceptible to confounding by disease severity and changes in care trajectories in the period preceding mortality. To counter this, comprehensive analysis adjustments were performed. Additionally, the number of involved GP clinics, assessing primary care fragmentation, may be less prone to confounding by severity; because GPs are generalists, there is rarely clinical indication to see multiple GPs even when complicated disease could entail appropriate specialized care across sectors. However, the individual’s disease severity and complexity may not be fully described by administrative data, so residual confounding might persist. The PIM categories represented well-defined inappropriate medication combinations but did not cover all suboptimal medical treatment. A potential limitation was that PIMs correlate with multimorbidity, which could have affected the estimates. PIM indicators may identify suboptimal clinical practice in a large cohort with average values between providers, but treatment applied at a patient level requires individual clinical interpretation.

### Implications

Care fragmentation remains a challenge in the provision of integrated care for patients with complex or comprehensive care needs. The patient’s need for both a close doctor-patient relationship and the need for highly specialized treatment at multiple sites can be conflicting. This may lead to high treatment burden, poor patient satisfaction, and adverse health outcomes. Our findings suggest that reducing care fragmentation by concentrating care on fewer providers, including frequent contact to the patient’s own GP and ensuring good coordination with fewer transitions, may be associated with better outcomes regardless of morbidity level. Only one in six of all contacts involved a hospital. Therefore, a large group of patients will not benefit from hospital interventions alone. Rather, primary care may provide the continuity of care needed to establish a coherent overview of the individual patient’s treatment and trajectories.

Interventions to improve the coordination of care for patients with multimorbidity have shown modest results and mixed effects on patient outcomes [50, 51]. Still, a large cluster-randomized trial in the UK has shown that better coordination of care improves the patient’s experience of care [52]. Integrating hospital care to improve care for patients with multimorbidity may also be feasible [53]. Sufficient resources, a strong focus on the patient-professional relationship, and technical solutions to support information flow may improve care continuity. Our results may be primarily generalizable to healthcare systems with a gate keeper function. However, it may be assumed that the basic elements of a patient pathway such as different contacts, transitions, and treatment responsibility are fundamental in most systems, and our finding may be interpreted more broadly. Nevertheless, more research is needed on individual and structural risk factors for care fragmentation and on effective intervention targeting patient pathway coordination, information flow, and relational continuity.

## Conclusions

Several clinical indicators of care fragmentation, including the number of contacts, healthcare providers, provider transitions, and hospital trajectories, were associated with higher morbidity level. High levels of all aspects of care fragmentation were associated with higher PIM rates and higher mortality even after adjusting for morbidity, demographics, and socioeconomic factors. Frequent contact to the usual provider, fewer transitions and better coordination were associated with better patient outcomes regardless of morbidity level.

### Supplementary Information

**Additional file 1**: **Methods S1.** Danish Multimorbidity Index definitions. **Methods S2.** Visualized example of a patient pathway. **Methods S3.** PIM criteria definitions according to the register-adapted STOPP/START criteria. **Table S1.** Incidence rate ratios of potentially inappropriate medication by care fragmentation measures. **Table S2.** Mortality hazard ratios by care fragmentation measures. **Table S3.** Incidence rate ratios of separate STOPP/START criteria by care fragmentation measures. **Figure S1.** Distribution of care fragmentation measures by number of conditions. **Figure S2.** Potentially inappropriate medication incidence rate ratios by care fragmentation measures and number of conditions. **Figure S3.** Mortality hazard ratios by care fragmentation measures and number of conditions.

## Data Availability

Dr. Anders Prior had full access to all the data in the study and takes responsibility for the integrity of the data and the accuracy of the data analysis. The dataset supporting the conclusions of this article are available at Statistics Denmark (https://www.dst.dk/en) and the Danish Health Data Authority (https://sundhedsdatastyrelsen.dk/da/english/). Data access is restricted to authorized research institutions by Danish law.

## Abbreviations

CI: Confidence interval
CIP: Cumulative incidence proportions
COCI: Continuity of Care Index
GP: General practitioner
HR: Hazard ratio
IRR: Incidence rate ratio
PIM: Potentially inappropriate medication
SECON: Sequential Continuity Index
UPC: Usual Provider of Care Index
