# Supplementary material for: Healthcare fragmentation, multimorbidity, potentially inappropriate medication, and mortality: a Danish nationwide cohort study
Source: BMC Med. 2023 Aug 15;21:305. doi: 10.1186/s12916-023-03021-3 (PMC10426166; doi:10.1186/s12916-023-03021-3)
Supplement: Supplementary file 1 — Additional file 1: Methods S1. Danish Multimorbidity Index definitions. Methods S2. Visualized example of a patient pathway. Methods S3. PIM criteria definitions according to the register-adapted STOPP/START criteria. Table S1. Incidence rate ratios of potentially inappropriate medication by care fragmentation measures. Table S2. Mortality hazard ratios by care fragmentation measures. Table S3. Incidence rate ratios of separate STOPP/START criteria by care fragmentation measures. Figure S1. Distribution of care fragmentation measures by number of conditions. Figure S2. Potentially inappropriate medication incidence rate ratios by care fragmentation measures and number of conditions. Figure S3. Mortality hazard ratios by care fragmentation measures and number of conditions. [file 12916_2023_3021_MOESM1_ESM.pdf]

Healthcare fragmentation, multimorbidity, potentially inappropriate medication, and mortality: a Danish nationwide cohort study

Supplementary methods, tables, and figures.

Contents:

Methods S1. Danish Multimorbidity Index definitions

Methods S2. Visualised example of a patient pathway

Table S1. Incidence rate ratios of potentially inappropriate medication by care fragmentation measures

Table S2. Mortality hazard ratios by care fragmentation measures

Table S3. Incidence rate ratios of separate STOPP/START criteria by care fragmentation measures

Figure S1. Distribution of care fragmentation measures by number of conditions

Figure S2. Potentially inappropriate medication incidence rate ratios by care fragmentation measures and number of conditions

Figure S3. Mortality hazard ratios by care fragmentation measures and number of conditions

Methods S3. PIM criteria definitions according to the register-adapted STOPP/START criteria

Methods S1. Danish Multimorbidity Index definitions

Category	Disease group	Coding definition	Diagnosis codes (ICD-10)	Diagnosis time frame	Drug codes (ATC)	Prescription time frame
**Circulatory system**	Hypertension	Diagnosis AND/OR prescriptions of antihypertensives, if not ischemic heart disease or heart failure (or kidney disease: only diuretics)	I10-I13, I15	Ever	C02, C04, C07, C08, C09, C03	Twice last year
	Dyslipidaemia	Diagnosis AND/OR drug prescription for lipid-lowering drugs if not ischemic heart disease.	E78	Last two years	C10	Twice last year
	Ischaemic heart disease	Diagnosis AND/OR prescription for antianginal drug	I20-I25	Ever	C01DA	Twice last year
	Atrial fibrillation	Diagnosis	I48	Ever		
	Heart failure	Diagnosis	I50	Ever		
	Peripheral artery occlusive disease	Diagnosis	I70-I74	Ever		
	Stroke	Diagnosis	I60-I64, I69	Ever		
**Endocrine system**	Diabetes mellitus	Diagnosis AND/OR prescription of antidiabetics	E10-E14	Ever	A10A, A10B	Twice last year
	Thyroid disorder	Diagnosis AND/OR prescription of thyroid therapy drugs	E00-E05, E061-E069, E07	Last two years	H03	Twice last year
	Gout	Diagnosis	E79, M10	Ever		
**Pulmonary system and allergy**	Chronic pulmonary disease	Prescription for obstructive airway disease drugs			R03	Twice last year
	Allergy	Prescription for non-sedative antihistamines AND/OR nasal antiallergics			R06AX, R06AE07, R06AE09, R01AC, R01AD	Twice last year
**Gastrointestinal system**	Ulcer/chronic gastritis	Diagnosis	K221, K25-K28, K293-K295	Ever		
	Chronic liver disease	Diagnosis	B16-B19, K70-K74, K766, I85	Ever		
	Inflammatory bowel disease	Diagnosis	K50-K51	Ever		
	Diverticular disease of intestine	Diagnosis	K57	Ever		
**Urogenital system**	Chronic kidney disease	Diagnosis	N03, N11, N18-N19	Ever		
	Prostate disorders	Diagnosis AND/OR prescription of prostate hyperplasia therapy drugs	N40	Ever	C02CA, G04C	Twice last year
**Musculoskeletal system**	Connective tissue disorders	Diagnosis	M05-M06, M08-M09, M30-M36, D86	Ever		
	Osteoporosis	Diagnosis AND/OR prescription for osteoporosis drugs	M80-M82	Ever	M05B, G03XC01, H05AA	Twice last year
	Painful condition	Repeated prescriptions of analgesics			N02A, N02BA51,N02BE, M01A, M02A	Four times last year
**Haematological system**	HIV/AIDS	Diagnosis	B20-B24	Ever		
	Anaemias	Diagnosis	D50-D53, D55-D59, D60-D61, D63-D64	Last two years		
**Cancers**	Cancer	Diagnosis	C00-C43, C45-C97	Last five years		
**Neurological system**	Vision problem	Diagnosis	H40, H25, H54	Ever		
	Hearing problem	Diagnosis	H90-H91, H931	Ever		
	Migraine	Diagnosis AND/OR prescription of specific anti-migraine drugs	G43	Last two years	N02C	Twice last year

	Epilepsy	Diagnosis AND prescription of anti-epileptics	G40-G47	Ever	N03	Twice last year
	Parkinson's disease	Diagnosis	G20-G22	Ever		
	Multiple sclerosis	Diagnosis	G35	Ever		
	Neuropathies	Diagnosis	G50-G64	Last two years		
**Mental health conditions**	Mood, stress-related, or anxiety disorders	Diagnosis	F32-F34, F40-F48	Last two years		
	Psychological distress	Prescription of antidepressants if not other mental disorder			N06A	Twice last year
	Alcohol problems	Diagnosis	F101-F109	Last two years		
	Substance abuse	Diagnosis	F11-F16, F18-F19	Last two years		
	Anorexia/bulimia	Diagnosis	F50	Last two years		
	Bipolar affective disorder	Diagnosis AND/OR prescription of lithium salts	F30-F31	Ever	N05AN	Twice last year
	Schizophrenia or schizoaffective disorder	Diagnosis	F20, F25	Ever		
	Dementia	Diagnosis AND/OR prescription of anti-dementia drugs	F00-F03, F051, G30	Ever	N06D	Twice last year

Abbreviations: ICD-10, International Classification of Diseases, version 10; ATC, Anatomical Therapeutic Chemical Classification System; HIV, human immunodeficiency virus; AIDS, acquired immunodeficiency syndrome.
ICD-10 diagnosis code recorded or redeemed prescription of ATC-coded drug registered within defined time frames in national health registers (the Danish National Patient Register, the Danish Psychiatric Central Register, and the Danish National Prescription Registry.)
[a]Prior A, Fenger-Grøn M, Larsen KK, et al. The association between perceived stress and mortality among people with multimorbidity: A prospective population-based cohort study. *Am J Epidemiol*. 2016;184(3):199-210. doi:10.1093/aje/kwv324

Methods S2. Visualised example of a patient pathway

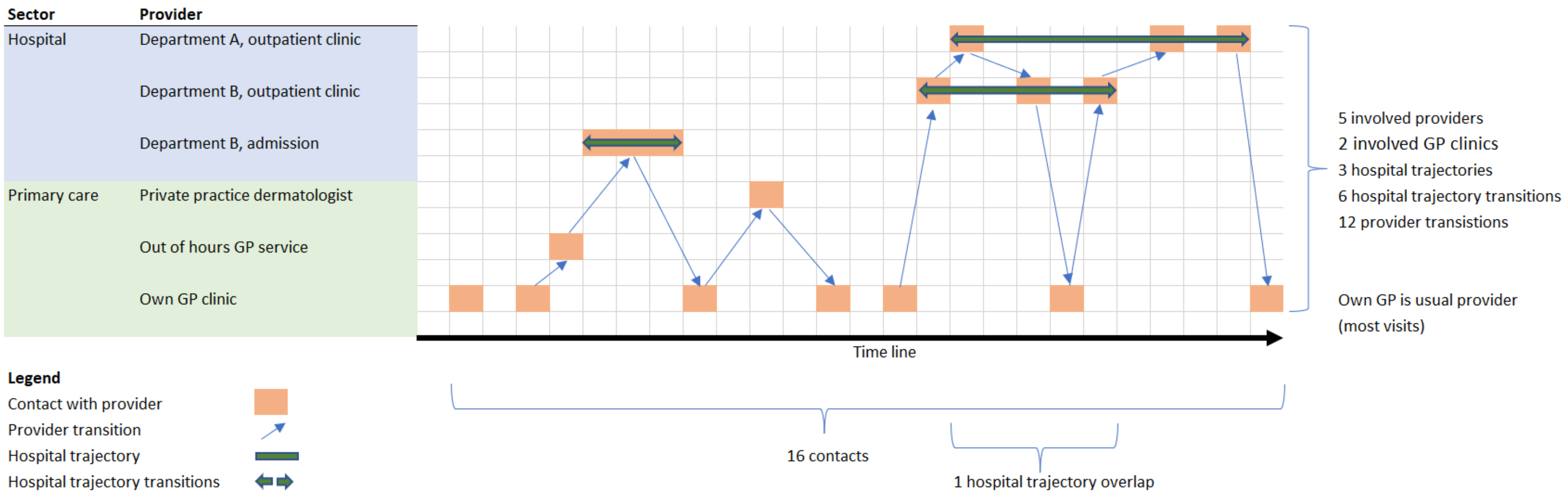

Usual provider index (UPC) = max(7/16; 1/16; 1/16; 4/16; 3/16) = 0.44
Continuity Of Care Index (COCI) = ((7^2+1^2+1^2+4^2+3^2)-1)/(16*(16-1)) = 0.31
Sequential Continuity Index (SECON) = (1+0+0+0+0+1+0+0+0+0+0+0+1+0)/(16-1) = 0.20
Known GP Index = 7/16 = 0.44

Table S1. Incidence rate ratios of potentially inappropriate medication by care fragmentation measures

Measure	Persons	Potentially inappropriate medication (PIM)					
	N (%)	PIM time (1000 yrs)	Time at risk (1000 yrs)	IRR*	95% CI	IRR**	95% CI
Involved providers							
0	558,761 (12.0)	26	478	1	Ref.	1	Ref.
1	1,168,709 (25.1)	131	1,096	1.82	(1.79,1.86)	1.51	(1.48,1.54)
2	950,672 (20.4)	150	901	2.30	(2.26,2.35)	1.76	(1.73,1.80)
3	669,717 (14.4)	136	636	2.81	(2.75,2.87)	1.99	(1.95,2.03)
4	439,971 (9.5)	111	418	3.38	(3.30,3.46)	2.21	(2.16,2.26)
5+	864,012 (18.6)	350	813	5.11	(5.00,5.21)	2.55	(2.50,2.60)
Involved GP clinics							
0	679,538 (14.6)	37	590	1	Ref.	1	Ref.
1	2,760,932 (59.4)	539	2,617	2.21	(2.18,2.25)	1.63	(1.60,1.65)
2	610,730 (13.1)	140	572	2.82	(2.76,2.88)	1.84	(1.80,1.88)
3	302,229 (6.5)	80	284	3.19	(3.11,3.27)	1.94	(1.89,1.99)
4	123,865 (2.7)	37	116	3.71	(3.58,3.85)	2.10	(2.03,2.17)
5+	174,548 (3.8)	70	161	5.02	(4.86,5.17)	2.28	(2.21,2.35)
Provider transitions							
0	1,727,470 (37.1)	157	1,574	1	Ref.	1	Ref.
1	431,562 (9.3)	49	405	1.25	(1.22,1.27)	1.20	(1.18,1.22)
2	548,650 (11.8)	92	521	1.56	(1.53,1.58)	1.35	(1.33,1.38)
3	313,008 (6.7)	55	297	1.68	(1.65,1.72)	1.44	(1.41,1.47)
4	309,222 (6.6)	68	294	1.91	(1.87,1.96)	1.53	(1.50,1.57)
5-9	766,470 (16.5)	213	729	2.33	(2.30,2.37)	1.71	(1.68,1.73)
10-19	430,859 (9.3)	185	408	3.29	(3.23,3.36)	1.92	(1.89,1.96)
20+	124,601 (2.7)	85	114	5.24	(5.06,5.42)	1.99	(1.92,2.06)
Total contacts							
0	558,761 (12.0)	26	478	1	Ref.	1	Ref.
1	332,401 (7.1)	21	307	1.37	(1.33,1.40)	1.29	(1.26,1.32)
2	310,107 (6.7)	24	289	1.56	(1.52,1.60)	1.42	(1.38,1.45)
3	290,378 (6.2)	26	272	1.73	(1.69,1.78)	1.52	(1.48,1.56)
4	272,206 (5.9)	28	256	1.89	(1.84,1.94)	1.61	(1.57,1.65)
5-9	1,067,263 (22.9)	150	1,012	2.39	(2.34,2.43)	1.87	(1.83,1.90)
10-19	1,083,715 (23.3)	264	1,034	3.57	(3.50,3.64)	2.34	(2.30,2.39)
20+	737,011 (15.8)	366	694	6.38	(6.24,6.52)	2.83	(2.77,2.90)
Hospital trajectory overlaps							
0	3,438,392 (73.9)	458	3,197	1	Ref.	1	Ref.
1	817,669 (17.6)	257	775	1.92	(1.89,1.94)	1.46	(1.44,1.48)
2	312,501 (6.7)	132	293	2.47	(2.42,2.52)	1.51	(1.48,1.54)
3	64,730 (1.4)	40	60	3.54	(3.38,3.70)	1.62	(1.55,1.69)
4	14,116 (0.3)	11	13	4.59	(4.17,5.06)	1.68	(1.53,1.85)
5+	4,434 (0.1)	5	4	6.18	(5.20,7.34)	1.71	(1.45,2.02)
Hospital trajectories							
0	3,438,392 (73.9)	458	3,197	1	Ref.	1	Ref.
1	622,157 (13.4)	180	592	1.81	(1.78,1.84)	1.44	(1.42,1.46)
2	274,764 (5.9)	100	259	2.17	(2.13,2.22)	1.50	(1.47,1.53)
3	141,936 (3.1)	60	133	2.43	(2.36,2.51)	1.52	(1.48,1.57)
4	71,571 (1.5)	36	67	2.86	(2.74,2.99)	1.58	(1.52,1.65)
5-9	89,117 (1.9)	58	81	3.54	(3.40,3.68)	1.55	(1.49,1.61)
10-19	13,014 (0.3)	11	11	5.23	(4.73,5.78)	1.56	(1.42,1.72)
20+	891 (0.0)	1	1	7.70	(5.25,11.31)	1.67	(1.16,2.41)
Hospital trajectory transitions							

0	3,448,408 (74.1)	461	3,206	1	Ref.	1	Ref.
1	217,924 (4.7)	70	209	1.97	(1.92,2.02)	1.53	(1.49,1.57)
2	426,717 (9.2)	121	404	1.76	(1.73,1.80)	1.40	(1.38,1.43)
3	93,616 (2.0)	35	89	2.31	(2.22,2.40)	1.54	(1.48,1.60)
4	17,0525 (3.7)	62	160	2.13	(2.07,2.19)	1.48	(1.44,1.52)
5+	294,652 (6.3)	154	272	2.94	(2.87,3.00)	1.52	(1.49,1.56)
Usual Provider of Care Index (UPC)							
1	494,140 (15.6)	82	469	1	Ref.	1	Ref.
0.75-0.99	1,078,017 (34.1)	304	1,028	1.46	(1.43,1.48)	1.2	(1.18,1.22)
0.50-0.74	1,150,510 (36.4)	309	1,090	1.53	(1.51,1.56)	1.25	(1.23,1.27)
0.25-0.49	412,095 (13.0)	107	386	1.69	(1.66,1.73)	1.37	(1.34,1.40)
0-0.24	25,433 (0.8)	6	23	1.90	(1.78,2.02)	1.49	(1.40,1.58)
Continuity Of Care Index (COCI)							
1	494,140 (15.6)	82	469	1	Ref.	1	Ref.
0.75-0.99	342,142 (10.8)	112	327	1.58	(1.55,1.62)	1.25	(1.23,1.28)
0.50-0.74	899,095 (28.5)	247	856	1.46	(1.43,1.48)	1.20	(1.18,1.22)
0.25-0.49	1,010,407 (32.0)	271	956	1.55	(1.53,1.58)	1.26	(1.24,1.28)
0-0.24	414,411 (13.1)	95	388	1.59	(1.56,1.62)	1.34	(1.31,1.37)
Sequential Continuity Index (SECON)							
1	494,140 (15.6)	82	469	1	Ref.	1	Ref.
0.75-0.99	560,632 (17.7)	160	532	1.47	(1.44,1.50)	1.21	(1.18,1.23)
0.50-0.74	1,136,502 (36.0)	311	1078	1.51	(1.48,1.53)	1.23	(1.21,1.25)
0.25-0.49	737,204 (23.3)	205	698	1.63	(1.60,1.66)	1.31	(1.28,1.33)
0-0.24	231,717 (7.3)	49	218	1.49	(1.45,1.52)	1.30	(1.27,1.34)
Known GP Index							
1	508,030 (16.1)	84	482	1	Ref.	1	Ref.
0.75-0.99	1,092,291 (34.6)	313	1,040	1.48	(1.45,1.50)	1.21	(1.19,1.23)
0.50-0.74	1,027,733 (32.5)	290	976	1.59	(1.57,1.62)	1.28	(1.26,1.30)
0.25-0.49	397,423 (12.6)	94	373	1.57	(1.54,1.60)	1.31	(1.29,1.34)
0-0.24	134,718 (4.3)	25	124	1.38	(1.34,1.43)	1.24	(1.21,1.28)

*Adjusted for age and sex, **Further adjusted for civil status, origin, educational attainment, income level, population density, and the 39 individual physical and mental conditions in the Danish Multimorbidity Index
IRR: Incidence rate ratio, Ref.: reference group, Yrs: years.

Table S2. Mortality hazard ratios by care fragmentation measures

**Measure**	**Mortality**					
	Deaths	CIP	HR*	95% CI	HR**	95% CI
Involved providers						
0	1,354	0.00242	1	Ref.	1	Ref.
1	6,370	0.00545	1.23	(1.16,1.31)	1.06	(1.00,1.12)
2	6,522	0.00686	1.20	(1.13,1.27)	0.98	(0.92,1.04)
3	6,048	0.00903	1.37	(1.29,1.45)	1.05	(0.99,1.12)
4	5,323	0.01210	1.65	(1.55,1.75)	1.17	(1.10,1.24)
5+	28,696	0.03321	3.53	(3.34,3.73)	1.89	(1.79,2.01)
Involved GP clinics						
0	1,926	0.00283	1	Ref.	1	Ref.
1	25,393	0.00920	1.37	(1.31,1.44)	1.06	(1.01,1.11)
2	8,974	0.01469	2.27	(2.16,2.38)	1.41	(1.34,1.48)
3	6,067	0.02007	2.83	(2.68,2.98)	1.59	(1.51,1.68)
4	3,353	0.02707	3.62	(3.42,3.83)	1.82	(1.72,1.93)
5+	8,600	0.04927	5.53	(5.26,5.82)	2.26	(2.14,2.38)
Provider transitions						
0	7,724	0.00447	1	Ref.	1	Ref.
1	1,778	0.00412	0.85	(0.81,0.90)	0.86	(0.82,0.91)
2	4,073	0.00742	1.08	(1.04,1.12)	0.98	(0.95,1.02)
3	2,499	0.00798	1.17	(1.12,1.23)	1.07	(1.02,1.12)
4	3,194	0.01033	1.25	(1.20,1.31)	1.09	(1.04,1.13)
5-9	11,506	0.01501	1.57	(1.53,1.62)	1.26	(1.22,1.29)
10-19	13,163	0.03055	2.50	(2.43,2.57)	1.66	(1.61,1.72)
20+	10,376	0.08327	5.90	(5.73,6.08)	2.80	(2.71,2.90)
Total contacts						
0	1,354	0.00242	1	Ref.	1	Ref.
1	702	0.00211	0.82	(0.75,0.90)	0.83	(0.76,0.91)
2	732	0.00236	0.82	(0.75,0.90)	0.83	(0.76,0.91)
3	763	0.00263	0.82	(0.75,0.90)	0.82	(0.75,0.90)
4	855	0.00314	0.87	(0.80,0.94)	0.86	(0.79,0.93)
5-9	5,134	0.00481	1.04	(0.98,1.10)	0.97	(0.91,1.03)
10-19	11,932	0.01101	1.60	(1.51,1.70)	1.27	(1.20,1.35)
20+	32,841	0.04456	4.19	(3.96,4.43)	2.26	(2.13,2.39)
Hospital trajectory overlaps						
0	20,026	0.00582	1	Ref.	1	Ref.)
1	15,489	0.01894	1.87	(1.83,1.91)	1.45	(1.42,1.48)
2	11,970	0.03830	3.37	(3.30,3.45)	2.13	(2.08,2.18)
3	4,714	0.07283	5.76	(5.58,5.95)	2.92	(2.82,3.02)
4	1,461	0.10350	7.96	(7.54,8.39)	3.49	(3.30,3.69)
5+	653	0.14727	11.7	(10.8,12.6)	4.07	(3.76,4.42)
Hospital trajectories						
0	20,026	0.00582	1	Ref.	1	Ref.
1	9,202	0.01479	1.55	(1.51,1.59)	1.29	(1.25,1.32)
2	6,357	0.02314	2.17	(2.11,2.24)	1.61	(1.56,1.66)
3	4,612	0.03249	2.84	(2.75,2.93)	1.93	(1.87,1.99)
4	3,338	0.04664	3.71	(3.57,3.85)	2.32	(2.23,2.41)
5-9	7,883	0.08846	6.32	(6.15,6.49)	3.38	(3.28,3.48)
10-19	2,665	0.20478	16.3	(15.6,16.9)	6.25	(5.98,6.54)
20+	230	0.25814	42.5	(37.3,48.4)	10.8	(9.48,12.4)
Hospital trajectory transitions						
0	20,206	0.00586	1	Ref.	1	Ref.
1	3,231	0.01483	1.50	(1.44,1.56)	1.17	(1.13,1.22)

2	6,473	0.01517	1.59	(1.54,1.63)	1.31	(1.28,1.35)
3	1,913	0.02043	2.10	(2.01,2.20)	1.46	(1.39,1.53)
4	4,363	0.02559	2.25	(2.18,2.32)	1.64	(1.59,1.70)
5+	18,127	0.06152	4.82	(4.72,4.92)	2.73	(2.67,2.79)
Usual Provider of Care Index (UPC)						
1	4,763	0.00964	1	Ref.	1	Ref.
0.75-0.99	19,003	0.01763	1.34	(1.29,1.38)	1.05	(1.02,1.09)
0.50-0.74	18,533	0.01611	1.46	(1.41,1.51)	1.19	(1.15,1.23)
0.25-0.49	7,960	0.01932	2.12	(2.05,2.20)	1.70	(1.64,1.76)
0-0.24	503	0.01978	3.79	(3.45,4.15)	2.59	(2.36,2.84)
Continuity Of Care Index (COCI)						
1	4,763	0.00964	1	Ref.	1	Ref.
0.75-0.99	7,849	0.02294	1.45	(1.40,1.51)	1.09	(1.05,1.13)
0.50-0.74	14,655	0.01630	1.31	(1.27,1.36)	1.05	(1.01,1.08)
0.25-0.49	16,861	0.01669	1.53	(1.48,1.58)	1.25	(1.21,1.29)
0-0.24	6,634	0.01601	2.07	(1.99,2.15)	1.70	(1.63,1.76)
Sequential Continuity Index (SECON)						
1	4,763	0.00964	1	Ref.	1	Ref.
0.75-0.99	11,578	0.02065	1.50	(1.45,1.56)	1.14	(1.10,1.18)
0.50-0.74	19,428	0.01709	1.44	(1.39,1.48)	1.14	(1.10,1.18)
0.25-0.49	12,399	0.01682	1.58	(1.53,1.64)	1.30	(1.25,1.34)
0-0.24	2,594	0.01119	1.49	(1.42,1.57)	1.39	(1.33,1.46)
Known GP Index						
1	4,907	0.00966	1	Ref.	1	Ref.
0.75-0.99	19,916	0.01823	1.35	(1.31,1.39)	1.05	(1.02,1.08)
0.50-0.74	16,867	0.01641	1.43	(1.39,1.48)	1.15	(1.12,1.19)
0.25-0.49	6,575	0.01654	1.92	(1.85,1.99)	1.65	(1.59,1.72)
0-0.24	2,497	0.01854	2.71	(2.58,2.84)	2.48	(2.36,2.60)

*Adjusted for age and sex, **Further adjusted for civil status, origin, educational attainment, income level, population density, and the 39 individual physical and mental conditions in the Danish Multimorbidity Index

CIP: Cumulative incidence proportion, HR: Hazard ratio, Ref.: reference group.

Table S3. Incidence rate ratios of separate STOPP/START criteria by care fragmentation measures

**Measure**	**START**				**STOPP**			
	PIM time (1,000 yrs)	Time at risk (1,000 yrs)	IRR*	95% CI	PIM time (1,000 yrs)	Time at risk (1,000 yrs)	IRR*	95% CI
Involved providers								
0	24	22	1	Ref.	1	478	1	Ref.
1	115	116	1.24	(1.22,1.25)	16	1,096	3.14	(3.04,3.24)
2	130	131	1.35	(1.33,1.36)	21	900	4.18	(4.04,4.32)
3	116	115	1.45	(1.43,1.46)	19	636	4.86	(4.69,5.04)
4	94	92	1.54	(1.52,1.56)	17	418	5.58	(5.36,5.81)
5+	293	267	1.70	(1.69,1.72)	57	813	7.21	(6.95,7.47)
Involved GP clinics								
0	35	31	1	Ref.	2	590	1	Ref.
1	465	457	1.29	(1.28,1.30)	74	2,617	3.16	(3.08,3.25)
2	118	113	1.39	(1.37,1.40)	22	572	3.95	(3.81,4.09)
3	67	64	1.43	(1.42,1.45)	13	284	4.23	(4.05,4.41)
4	31	28	1.50	(1.48,1.53)	6	116	5.03	(4.74,5.34)
5+	57	49	1.61	(1.58,1.63)	13	161	5.98	(5.67,6.31)
Provider transitions								
0	139	138	1	Ref.	17	1,573	1	Ref.
1	44	43	1.12	(1.11,1.13)	5	405	1.28	(1.24,1.33)
2	79	80	1.18	(1.17,1.19)	13	521	1.77	(1.72,1.82)
3	48	47	1.24	(1.22,1.25)	7	297	1.76	(1.70,1.83)
4	58	58	1.26	(1.25,1.27)	10	294	2.10	(2.02,2.18)
5-9	181	177	1.35	(1.34,1.36)	32	729	2.48	(2.42,2.55)
10-19	154	143	1.46	(1.45,1.47)	31	408	3.01	(2.91,3.12)
20+	68	56	1.54	(1.52,1.56)	16	114	3.52	(3.32,3.72)
Total contacts								
0	24	22	1	Ref.	1	478	1	Ref.
1	20	19	1.16	(1.15,1.18)	1	307	1.69	(1.62,1.77)
2	22	21	1.22	(1.20,1.24)	2	289	2.16	(2.07,2.26)
3	24	23	1.27	(1.25,1.28)	2	272	2.77	(2.65,2.90)
4	25	25	1.30	(1.28,1.32)	2	256	2.97	(2.84,3.11)
5-9	135	135	1.40	(1.39,1.42)	15	1,012	4.10	(3.96,4.23)
10-19	227	229	1.56	(1.55,1.58)	36	1,034	6.62	(6.40,6.85)
20+	295	270	1.73	(1.71,1.75)	71	693	9.98	(9.61,10.36)
Hospital trajectory overlaps								
0	398	392	1	Ref.	60	3,197	1	Ref.
1	218	210	1.24	(1.23,1.25)	39	775	1.82	(1.77,1.86)
2	110	102	1.28	(1.26,1.29)	22	292	2.03	(1.96,2.11)
3	33	29	1.35	(1.32,1.37)	7	60	2.35	(2.18,2.54)
4	9	7	1.39	(1.34,1.45)	2	13	2.61	(2.22,3.06)
5+	4	3	1.50	(1.40,1.60)	1	4	2.38	(1.79,3.17)
Hospital trajectories								
0	398	392	1	Ref.	60	3,197	1	Ref.
1	153	149	1.23	(1.22,1.24)	27	592	1.80	(1.76,1.85)
2	84	80	1.26	(1.25,1.27)	16	259	1.91	(1.84,1.99)
3	50	47	1.28	(1.27,1.30)	10	133	2.04	(1.94,2.15)
4	30	27	1.32	(1.30,1.34)	6	67	2.06	(1.92,2.21)
5-9	48	41	1.33	(1.31,1.36)	10	81	2.24	(2.10,2.39)
10-19	9	7	1.42	(1.36,1.48)	2	11	2.66	(2.25,3.14)
20+	1	0.4	1.48	(1.27,1.72)	0.2	1	2.63	(1.40,4.95)

Hospital trajectory transitions								
0	400	394	1	Ref.	61	3,206	1	Ref.
1	59	58	1.26	(1.24,1.27)	11	209	2.10	(2.01,2.19)
2	103	99	1.22	(1.21,1.23)	18	404	1.67	(1.62,1.72)
3	29	28	1.28	(1.26,1.30)	6	89	2.08	(1.95,2.22)
4	53	50	1.26	(1.24,1.27)	10	160	1.78	(1.70,1.87)
5+	128	114	1.3	(1.29,1.32)	26	272	2.06	(1.98,2.14)
Usual Provider of Care Index (UPC)								
1	69	72	1	Ref.	13	469	1	Ref.
0.75-0.99	250	246	1.11	(1.10,1.12)	54	1,027	1.3	(1.27,1.34)
0.50-0.74	265	250	1.18	(1.17,1.19)	44	1,090	1.18	(1.15,1.22)
0.25-0.49	94	85	1.27	(1.25,1.28)	13	386	1.2	(1.16,1.25)
0-0.24	5	4	1.36	(1.32,1.41)	1	23	1.17	(1.05,1.31)
Continuity Of Care Index (COCI)								
1	69	72	1	Ref.	13	469	1	Ref.
0.75-0.99	89	89	1.11	(1.10,1.12)	23	327	1.57	(1.51,1.63)
0.50-0.74	206	201	1.13	(1.12,1.14)	41	856	1.22	(1.18,1.26)
0.25-0.49	233	220	1.19	(1.18,1.20)	38	956	1.19	(1.15,1.22)
0-0.24	85	77	1.26	(1.25,1.27)	11	387	1.1	(1.06,1.14)
Sequential Continuity Index (SECON)								
1	69	72	1	Ref.	13	469	1	Ref.
0.75-0.99	130	129	1.11	(1.10,1.12)	30	532	1.38	(1.34,1.43)
0.50-0.74	262	253	1.15	(1.14,1.16)	49	1,078	1.22	(1.19,1.26)
0.25-0.49	178	165	1.22	(1.21,1.23)	27	698	1.21	(1.17,1.25)
0-0.24	43	40	1.24	(1.22,1.25)	5	218	1.03	(0.99,1.08)
Known GP Index								
1	71	75	1	Ref.	13	482	1	Ref.
0.75-0.99	257	253	1.12	(1.11,1.13)	56	1,040	1.32	(1.28,1.36)
0.50-0.74	249	235	1.20	(1.19,1.21)	41	976	1.21	(1.17,1.24)
0.25-0.49	83	76	1.23	(1.22,1.25)	11	373	1.13	(1.09,1.17)
0-0.24	23	20	1.20	(1.18,1.22)	3	124	1.12	(1.06,1.17)

*Adjusted for age, sex, civil status, origin, educational attainment, income level, population density, and the 39 individual physical and mental conditions in the Danish Multimorbidity Index

IRR: Incidence rate ratio

Definitions in:

Potentially inappropriate medications (PIMs): frequency and extent of GP-related variation in PIMs: a register-based cohort study. Ribe et al., BMJ Open, 2021

STOPP/START criteria for potentially inappropriate prescribing in older people: Version 2. O'Mahony et al. 2015

Figure S1. Distribution of care fragmentation measures by number of conditions

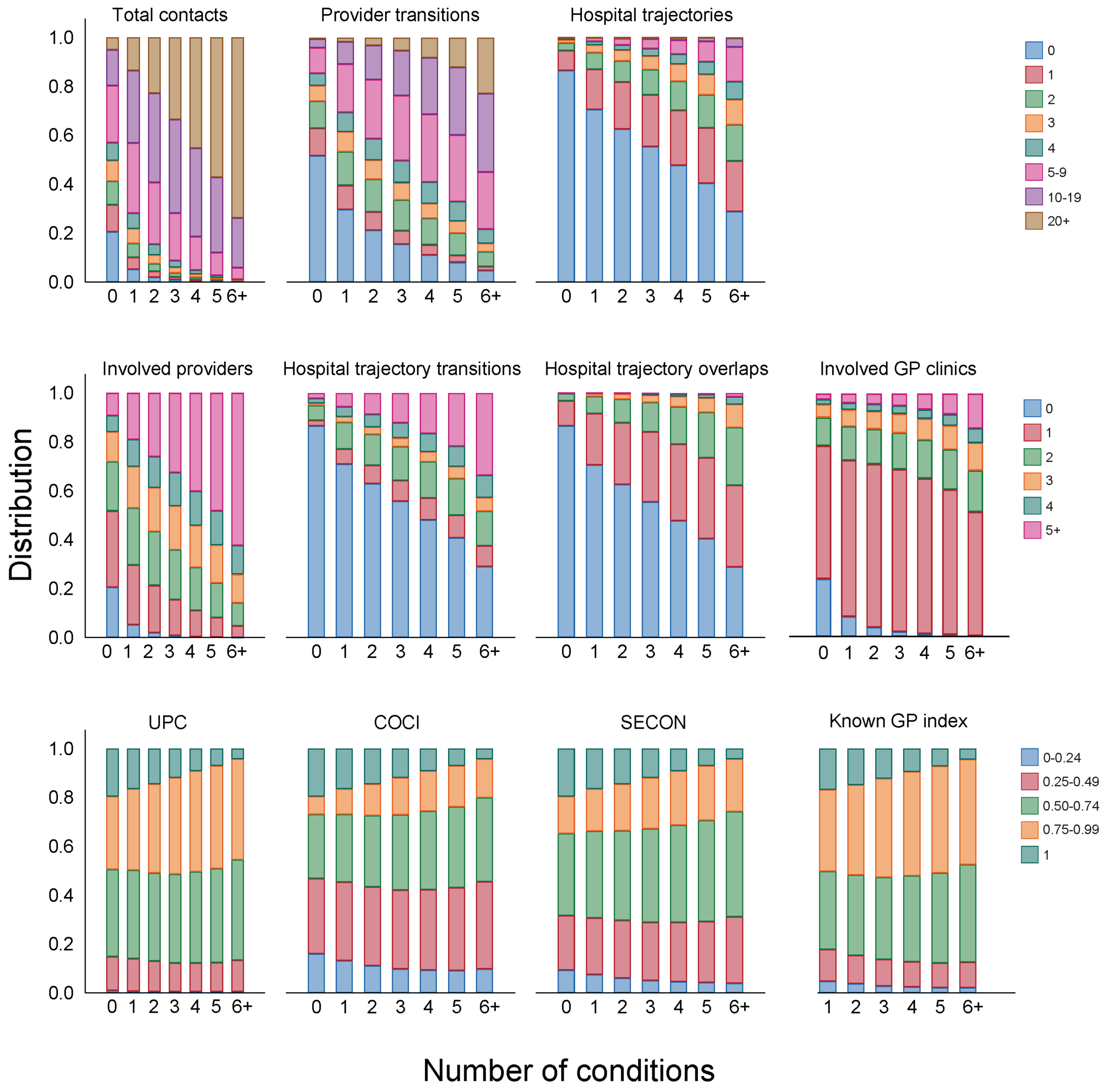

The values of the colours are stated to the right.

UPC: Usual Provider of Care Index, SECON: Sequential Continuity Index, COCI: Continuity of Care Index, GP: General practitioner

Figure S2. Potentially inappropriate medication incidence rate ratios by care fragmentation measures and number of conditions

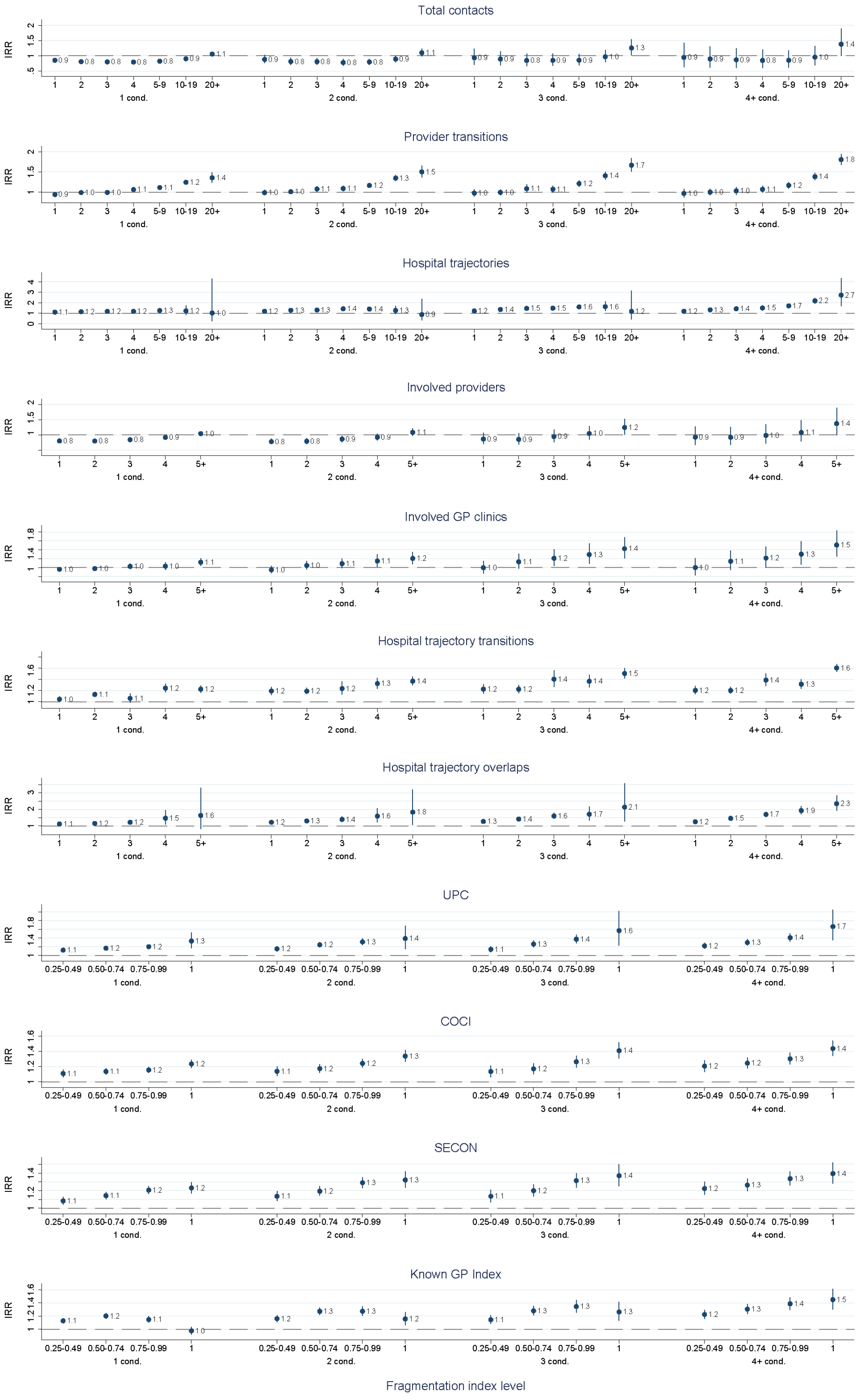

UPC: Usual Provider of Care Index, SECON: Sequential Continuity Index, COCI: Continuity of Care Index, GP: General practitioner, Cond.: Conditions

Figure S3. Mortality hazard ratios by care fragmentation measures and number of conditions

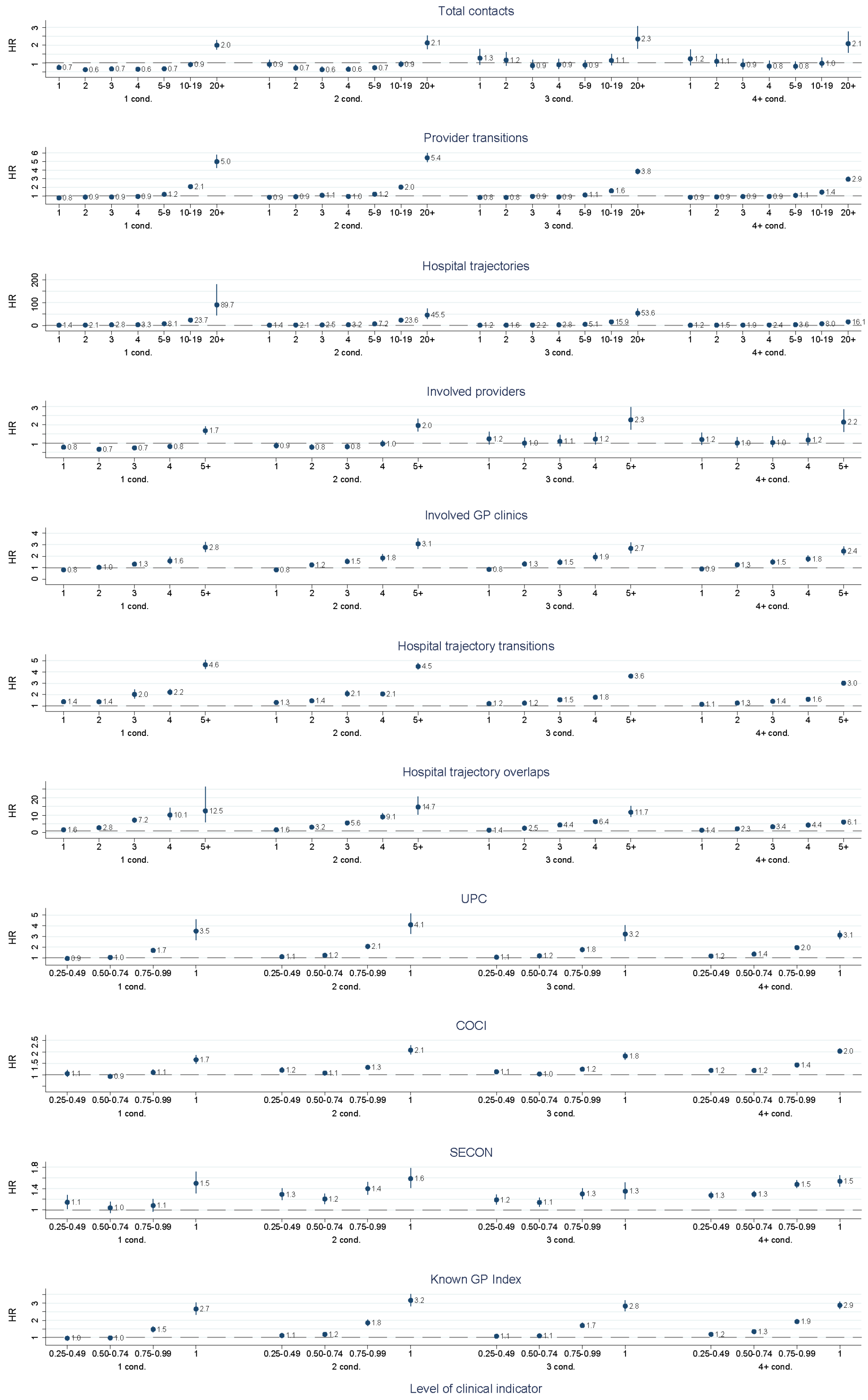

UPC: Usual Provider of Care Index, SECON: Sequential Continuity Index, COCI: Continuity of Care Index, GP: General practitioner, Cond.: Conditions

Methods S3. PIM criteria definitions according to the register-adapted STOPP/START criteria

ID	Description	A<br>ICD-10<br>*Atrial fibrillation*	B<br>ATC<br>*Vitamin K antagonists, direct thrombin inhibitors or factor Xa inhibitors*	Risk	Code
START A1	Start vitamin K antagonists or direct thrombin inhibitors or factor Xa inhibitors in the presence of chronic atrial fibrillation.	I48*	B01AA*, B01AE*, B01AF*	A if CHADVASc >=2	A & !B if CHADVASc >=2

ID	Description	A<br>ATC<br>*Vit K antagonists, direct thrombin inhibitors or factor Xa inhibitors*	B<br>ICD-10<br>*Coronary, cerebral or peripheral vascular disease*	C<br>Procedure codes<br>*Coronary implants (CABG or PCI)*	D<br>ATC<br>*Antiplatelet agents*	Risk	Code
START A3	Start antiplatelet therapy (acetylsalisylic acid or clopidogrel or prasugrel or ticagrelor) with a documented history of coronary, cerebral or peripheral vascular disease.	B01AA*, B01AE*, B01AF*	I20*, I21*, I22*, I23*, I24*, I25*, E10.5, E11.5, E14.5, I70.2, I73.9, I74.3, I63*, I64*, I65*, I66*, G45*	KFN*, KZFX01	B01AC*	!A & (B | C)	[!A & (B | C)] & !D

ID	Description	A<br>ICD-10<br>*Coronary, cerebral or peripheral vascular disease*	B<br>Procedure codes<br>*Coronary implants (CABG or PCI)*	C<br>ICD-10<br>*Chronic kidney disease*	D<br>ICD-10<br>*Rhabdomyolysis*	E<br>ATC<br>*Statins*	Risk	Code
START A5	Start statin therapy with a documented history of coronary, cerebral or peripheral vascular disease, unless the patient's status is end-of-life or age is > 85 years.	I20*, I21*, I22*, I23*, I24*, I25*, E10.5, E11.5, E14.5, I70.2, I73.9, I74.3, I63*, I64*, I65*, I66*, G45*	KFN*, KZFX01	N03*, N011*, N18*, N19*	M62.8, T79.6	C10AA*, C10B*	(A | B) & [!(C | [D within 6 months])]	{(A | B) & [!(C | [D within 6 months])]} & !E

ID	Description	A<br>ICD-10<br>*Heart failure*	B<br>ATC<br>*Angiotensin-converting-enzyme inhibitors or angiotensin II receptor blockers*	Risk	Code
START A6	Start ACE inhibitor with systolic heart failure and/or documented coronary artery disease.	I50*, I11.0, I13.0, I13.2	C09A*, C09B*, C09C*, C09D*	A	A & !B

ID	Description	A<br>ICD-10<br>*Ischaemic heart disease*	B<br>Procedure codes<br>*Coronary implants (CABG or PCI)*	C<br>ICD-10<br>*Heart failure*	D<br>ATC<br>*Beta blockers*	Risk	Code
START A7	Start beta-blocker with ischaemic heart disease.	I20*, I21*, I22*, I24*, I25*	KFN*, KZFX01	I50*, I11.0, I13.0, I13.2	C07*	(A | B) & !C	[(A | B) & !C] & !D

ID	Description	A<br>ICD-10<br>*Heart failure*	B<br>ATC<br>*Beta blockers (bisoprolol, nebivolol, metoprolol, or carvedilol)*	Risk	Code
START A8	Start appropriate beta-blocker (bisoprolol, nebivolol, metoprolol or carvedilol) with stable systolic heart failure	I50*, I11.0, I13.0, I13.2	C07AB02, C07AB07, C07AB12, C07AG02, C07BB02, C07BB07, C07BB12, C07BB52, C07CB02, C07FB02, C07FB07, C07FB12	A	A & !B

ID	Description	A<br>ICD-10<br>*Asthma or chronic obstructive pulmonary disease*	B<br>ATC<br>*Beta-2 agonists (inhalation) or antimuscarinic bronchodilators*	Risk	Code

**START B1**	Start regular inhaled b2 agonist or antimuscarinic bronchodilator (e.g. ipratropium, tiotropium) for mild to moderate asthma or COPD.	J40, J41*, J42, J43*, J44*, J45*, J46	R03AC*, R03AK04, R03AK05, R03AK06, R03AK07, R03AK08, R03AK09, R03AK10, R03AK11, R03AK12, R03AK13 ,R03AL*, R03BB*	A	A & !B

**ID**	**Description**	**A**<br>ICD-10<br>*Alzheimer's dementia or Lewy body dementia*	**B**<br>ATC<br>*Acetylcholinesterase inhibitors*	**Risk**	**Code**
**START C3**	Start acetylcholinesterase inhibitor (e.g. donepezil, rivastigmine, galantamine) for mild-moderate Alzheimer's dementia or Lewy Body dementia (rivastigmine).	G30*, F00*, G31.8	N06DA*	A	A & !B

**ID**	**Description**	**A**<br>ICD-10<br>*Diabetes mellitus*	**B**<br>ICD-10<br>*Proteinuria*	**C**<br>ICD-10<br>*Diabetes mellitus with renal manifestations*	**D**<br>ATC<br>*Angiotensin-converting-enzyme inhibitors or angiotensin II receptor blockers*	**Risk**	**Code**
**START F1**	Start ACE inhibitor or Angiotensin Receptor Blocker (if intolerant of ACE inhibitor) in diabetes with evidence of renal disease i.e. dipstick proteinuria or microalbuminuria (>30mg/24 hours) with or without serum biochemical renal impairment.	E10*, E11*, E12*, E13*, E14*	R80	E10.2, E11.2, E12.2, E13.2, E14.2, N08.3	C09A*, C09B*, C09C*, C09D*	(A & B (B later than A)) | C	((A & B (B later than A)) | C) & !D

**ID**	**Description**	**A**<br>ICD-10<br>*Prostatism*	**B**<br>ICD-10<br>*Urinary retention*	**C**<br>Procedure codes<br>*Prostatectomy*	**D**<br>ICD-10<br>*Heart failure*	**E**<br>ICD-10<br>*Ortostatic hypotension or syncope*	**F**<br>ATC<br>*Alpha-1 blockers or 5-alpha-reductase inhibitors*	**Risk**	**Code**
**START G1**	Start alpha-1 receptor blocker with symptomatic prostatism, where prostatectomy is not considered necessary.<br><br>Start 5-alpha reductase inhibitor with symptomatic prostatism, where prostatectomy is not considered necessary.	N40*	R33*	KKCC10, KKCC11, KKCC20, KKCC21, KKEC00, KKEC01, KKEC10, KKEC20	I50*, I11.0, I13.0, I13.2	I95.1, R55*	C02CA, G04CA*, G04CB*	(Male sex & [A | (B within 6 months)]) & !(C | D | [E within 6 months])	{(Male sex & [A | (B within 6 months)]) & !(C | D | [E within 6 months])} & !F

**ID**	**Description**	**A**<br>ATC<br>*Verapamil (and combinations) and diltiazem*	**B**<br>ATC<br>*Beta blockers*	**Risk**	**Code**
**STOPP B3**	Stop beta-blocker in combination with verapamil or diltiazem (risk of heart block)	C08DA01, C09BB10, C08DA51, C08DB01	C07*	All	A & B (in combination)

**ID**	**Description**	**A**<br>ICD-10<br>*Bradycardia*	**B**<br>ICD-10<br>*AV block or other heart blocks*	**C**<br>ATC<br>*Beta blockers*	**Risk**	**Code**
**STOPP B4**	Stop beta-blocker with bradycardia (<50/min), type II heart block or complete heart block (risk of complete heart block, asystole)	I49.5, R00.1	I44.1, I44.2, I44.3, I45.5, I45.9, Q24.6	C07*	(A within 6 months) | B	[(A within 6 months) | B] & C

**ID**	**Description**	**A**<br>ICD-10<br>*Supraventricular tachyarrhythmias*	**B**<br>ATC<br>*Beta blockers, digoxin, verapamil, or diltiazem*	**C**<br>ATC<br>*Amiodarone*	**Risk**	**Code**
**STOPP B5**	Stop amiodarone as first-line antiarrhythmic therapy in supraventricular tachyarrhythmias (higher risk of side-effects than beta-blockers, digoxin, verapamil or diltiazem)	I47.1, I48*	C01AA05, C07*, C08DA01, C08DB01	C01BD01	A & !B (within 3 years)	A & !B (within 3 years) & C

**ID**	**Description**	**A**<br>ICD-10	**B**<br>ICD-10	**C**<br>ICD-10	**D**<br>ATC	**E**<br>ATC	**Risk**	**Code**

		Hypertension	*Heart failure*	*Chronic kidney disease*	*Potassium-sparing agents, thiazides, beta blockers, calcium channel blockers, agents acting on the renin-angiotensin system*	*Loop diuretics*		
**STOPP B6**	Loop diuretic as first-line treatment for hypertension (safer, more effective alternatives available).	I10*, I15*	I50*, I11.0, I13.0, I13.2	N03*, N011*, N18*, N19*	C03D*, C03AA*, C07*, C08*, C09*	C03C*, C03EB*	A & ![B | C | (D within 3 years)]	(A & ![B | C | (D within 3 years)]) & E

**ID**	**Description**	**A**<br>ICD-10<br>*Heart failure*	**B**<br>ATC<br>*Phosphodiesterase-5 selective inhibitors*	**Risk**	**Code**
**STOPP B13**	Stop phosphodiesterase type-5 inhibitors (e.g. sildenafil, tadalafil, vardenafil) in severe heart failure characterized by hypotension i.e. systolic BP < 90 mmHg, or concurrent nitrate therapy for angina (risk of cardiovascular collapse)	I50*, I11.0, I13.0, I13.2	G04BE03, G04BE08, G04BE09, G04BE10	A	A & B

**ID**	**Description**	**A**<br>ICD-10<br>*Bleeding diathesis*	**B**<br>ICD-10<br>*Recent gastrointestinal bleeding*	**C**<br>ICD-10<br>*Cerebral hemorrhage*	**D**<br>ATC<br>*Antiplatelet agents, vitamin K antagonists, direct thrombin inhibitors, or factor Xa inhibitors*	**Risk**	**Code**
**STOPP C3**	Acetylsalisylic acid, clopidogrel, dipyridamole, vitamin K antagonists, direct thrombin inhibitors or factor Xa inhibitors with concurrent significant bleeding risk, i.e. uncontrolled severe hypertension, bleeding diathesis, recent non-trivial spontaneous bleeding) (high risk of bleeding).	D66*, D67*, D68.0*, D69.3	K92.0, K92.1, K92.2	I60*, I61*, I62*	B01AA*, B01AC*, B01AE*, B01AF*	A | ((B | C) within 6 months)	(A | ((B | C) within 6 months)) & D

**ID**	**Description**	**A**<br>ICD-10<br>*Ischaemic cerebrovascular disease*	**B**<br>ICD-10<br>*Acute coronary syndrome*	**C**<br>Procedure codes<br>*Coronary implants (CABG or PCI)*	**D**<br>ICD-10<br>*Carotid stenosis*	**E**<br>ATC<br>*Acetylsalicylic acid, carbasalate calcium, or combinations of antiplatelet agents*	**F**<br>ATC<br>*Clopidogrel*	**Risk**	**Code**
**STOPP C4**	Stop acetylsalisylic acid plus clopidogrel as secondary stroke prevention, unless the patient has a coronary stent(s) inserted in the previous 12 months or concurrent acute coronary syndrome or has a high grade symptomatic carotid arterial stenosis (no evidence of added benefit over clopidogrel monotherapy)	I63*, I64*, G45*	I20.0, I21*, I22*	KFN*, KZFX01	I65.2	B01AC06, B01AC08, B01AC56, B01AC30	B01AC04	A & {!([B | C] within 12 months) | !([D within 3 months] & [A within 3 months])}	[A & {!([B | C] within 12 months) | !([D within 3 months] & [A within 3 months])}] & (E & F in combination)

**ID**	**Description**	**A**<br>Procedure codes<br>*Coronary implants (CABG or PCI)*	**B**<br>ICD-10<br>*Acute coronary syndrome*	**C**<br>ATC<br>*Vitamin K antagonists, direct thrombin inhibitors, or factor Xa inhibitors*	**D**<br>ATC<br>*Antiplatelet agents*	**Risk**	**Code**
**STOPP C6**	Stop antiplatelet agents with vitamin K antagonist, direct thrombin inhibitor or factor Xa inhibitors in patients with stable coronary, cerebrovascular or peripheral arterial disease (No added benefit from dual therapy).	KFN*, KZFX01	I20.0, I21*, I22*	B01AA*, B01AE*, B01AF*	B01AC*	!(A | B) within 12 months	(![A | B] within 12 months) & (C & D (in combination))

**ID**	**Description**	**A**<br>ICD-10<br>*Deep venous thrombosis*	**B**<br>ICD-10<br>*History of pulmonary embolus*	**C**<br>ICD-10<br>*Atrial fibrillation*	**D**<br>Procedure codes<br>*Mechanical prosthetic heart valves*	**E**<br>ATC<br>*Vitamin K antagonists, direct thrombin inhibitors, or factor Xa inhibitors*	**Risk**	**Code**
**STOPP C8**	Stop vitamin K antagonist, direct thrombin inhibitor or factor Xa inhibitors for first deep venous thrombosis without continuing provoking risk factors (e.g. thrombophilia) for > 6 months, (no proven added benefit)	I80*	I26*	I48*	KFCA60, KFGE00, KFJF00, KFKD00, KFKD51, KFMD00, KFMD54	B01AA*, B01AE*, B01AF*	(A for the first time but more than 6 months ago) & !(B | C | D)	[(A for the first time but more than 6 months ago) & !(B | C | D)] & E

ID	Description	A<br>ICD-10<br>*Pulmonary embolus*	B<br>ICD-10<br>*History of deep venous thrombosis*	C<br>ICD-10<br>*Atrial fibrillation*	D<br>Procedure codes<br>*Mechanical prosthetic heart valves*	E<br>ATC<br>*Vitamin K antagonists, direct thrombin inhibitors, or factor Xa inhibitors*	Risk	Code
**STOPP C9**	Stop vitamin K antagonist, direct thrombin inhibitor or factor Xa inhibitors for first pulmonary embolus without continuing provoking risk factors (e.g. thrombophilia) for > 12 months (no proven added benefit)	I26*	I80*	I48*	KFCA60, KFGE00, KFJF00, KFKD00, KFKD51, KFMD00, KFMD54	B01AA*, B01AE*, B01AF*	(A for the first time but more than 12 months ago) & !(B | C | D)	[(A for the first time but more than 12 months ago) & !(B | C | D)] & E

ID	Description	A<br>ATC<br>*Vitamin K antagonists, direct thrombin inhibitors, or factor Xa inhibitors*	B<br>ATC<br>*Nonsteroidal anti-inflammatory drugs*	Risk	Code
**STOPP C10**	Stop NSAID and vitamin K antagonist, direct thrombin inhibitor or factor Xa inhibitors in combination (risk of major gastrointestinal bleeding).	B01AA*, B01AE*, B01AF*	M01A*, M01BA*, N02BA*	All	A & B (in combination)

ID	Description	A<br>ICD-10<br>*Dementia*	B<br>ICD-10<br>*Narrow angle glaucoma*	C<br>ICD-10<br>*Cardiac conduction abnormalities*	D<br>ICD-10<br>*Prostatism or urinary retention*	E<br>ATC<br>*Tricyclic antidepressants*	Risk	Code
**STOPP D1**	Stop tricyclic antidepressants (TCAs) with dementia, narrow angle glaucoma, cardiac conduction abnormalities, prostatism, or prior history of urinary retention (risk of worsening these conditions).	F00*, F01*, F02*, F03, G30*, G31.0, G31.1, G31.8	H40.2	I44*, I45*	N40*, R33*	N06AA*, N06CA01, N06CA02	A | B | C | (D within 6 months)	(A | B | C | (D within 6 months)) & E

ID	Description	A<br>ICD-10<br>*Prostatism or urinary retention*	B<br>ATC<br>*Chlorpromazine, clozapine, flupentixol, fluphenzine, pipotiazine, promazine, or zuclopenthixol*	Risk	Code
**STOPP D3**	Stop neuroleptics with moderate-marked antimuscarinic/anticholinergic effects (chlorpromazine, clozapine, flupenthixol, fluphenzine, pipothiazine, promazine, zuclopenthixol) with a history of prostatism or previous urinary retention (high risk of urinary retention).	N40*, R33*	N05AA01, N05AA03, N05AB02, N05AC04, N05AF01, N05AF05, N05AH02	(A within 6 months)	(A within 6 months) & B

ID	Description	A<br>ATC<br>*Benzodiazepines*	Risk	Code
**STOPP D5**	Stop benzodiazepines for ≥ 4 weeks (no indication for longer treatment; risk of prolonged sedation, confusion, impaired balance, falls, road traffic accidents; all benzodiazepines should be withdrawn gradually if taken for more than 4 weeks as there is a risk of causing a benzodiazepine withdrawal syndrome if stopped abruptly).	N03AE*, N05BA*, N05CD*, N05CF*	All	A, if more than 30 DDDs within 3 months

ID	Description	A<br>ICD-10<br>*Parkinsonism or Lewy Body dementia*	B<br>ATC<br>*Antipsychotics (excluding quetiapine, clozapine and lithium)*	Risk	Code
**STOPP D6**	Stop antipsychotics (i.e. other than quetiapine or clozapine) in those with parkinsonism or Lewy Body Disease (risk of severe extra-pyramidal symptoms)	G20, G21*, G23.1, G23.2, F02.3, G31.8	N05AA*, N05AB*, N05AC*, N05AD*, N05AE*, N05AF*, N05AG*, N05AH01, N05AH03, N05AH05, N05AH06, N05AK*, N05AL*, N05AX*	A	A & B

ID	Description	A<br>ICD-10<br>*Delirium*	B<br>ICD-10<br>*Dementia*	C<br>ATC<br>*Anticholinergic agents*	Risk	Code

<table>
<tr><td>STOPP D8</td><td>Stop anticholinergics/antimuscarinics in patients with delirium or dementia (risk of exacerbation of cognitive impairment).</td><td>F05*</td><td>F00*, F01*, F02*, F03*, G30*, G31.0, G31.1, G31.8</td><td>G04BD01, G04BD02, G04BD03, G04BD04, G04BD05, G04BD06, G04BD07, G04BD08, G04BD09, G04BD10, G04BD11, N03AF01, N03AF02, N05AA01, N05AA02, N05AA04, N05AB02, N05AB03, N05AB04, N05AC01, N05AC02, N05AF04, N05AG02, N05AH03, N05AH02, N05BB01, N05BB51, N06AA*, R01AX03, R03AC06, R03AL01, R03AL02, R03AL03, R03AL05, R03AL07, R03AL09, R03BB01, R03BB04, R03BB05, R03BB06, R03BB07, R06AA*, R06AB*, R06AC*, R06AD* , R06AE01, R06AE03, R06AE04, R06AE05, R06AE06, R06AE51, R06AE53, R06AE55, R06AX01, R06AX02, R06AX03, R06AX04, R06AX05, R06AX08, R06AX09, R06AX13, R06AX15, R06AX16, R06AX17,</td><td>(A within 6 months) | B</td><td>((A within 6 months) | B) & C</td></tr>
</table>

<table>
<tr><th>ID</th><th>Description</th><th>A
ICD-10
Dementia</th><th>B
ICD-10
Schizophrenic disorders or psychotic disorders</th><th>C
ICD-10
Sleeping disorders</th><th>D
ATC
Antipsychotics</th><th>Risk</th><th>Code</th></tr>
<tr><td>STOPP D9</td><td>Stop neuroleptic antipsychotic in patients with behavioral and psychological symptoms of dementia (BPSD) unless symptoms are severe and other non-pharmacological treatments have failed (increased risk of stroke).</td><td>F00*, F01*, F02*, F03*, G30*, G31.0, G31.1, G31.8</td><td>F20*, F25*, F29*</td><td>F51.0, G47.0</td><td>N05AA*, N05AB*, N05AC*, N05AD*, N05AE*, N05AF*, N05AG*, N05AH*, N05AK*, N05AL*, N05AX*</td><td>A & !B & !(C within 6 months)</td><td>(A & !B & !(C within 6 months)) & D</td></tr>
</table>

<table>
<tr><th>ID</th><th>Description</th><th>A
ICD-10
Dementia</th><th>B
ICD-10
Bradycardia</th><th>C
ICD-10
Heart block</th><th>D
ICD-10
Syncope</th><th>E
ATC
Beta blockers, digoxin, diltiazem, or verapamil</th><th>F
ATC
Acetylcholinesterase inhibitors</th><th>Risk</th><th>Code</th></tr>
<tr><td>STOPP D11</td><td>Stop acetylcholinesterase inhibitors with a known history of persistent bradycardia (< 60 beats/min.), heart block or recurrent unexplained syncope or concurrent treatment with drugs that reduce heart rate such as beta-blockers, digoxin, diltiazem, verapamil (risk of cardiac conduction failure, syncope and injury).</td><td>F00*, F01*, F02*, F03, G30*, G31.0, G31.1, G31.8</td><td>I49.5, R00.1</td><td>I44.1, I44.2, I44.3, I45.5, I45.9 , Q24.6</td><td>R55*</td><td>C01AA05, C07*, C08DA01, C08DA51, C08DB01, C09BB10</td><td>N06DA*</td><td>A & (B (within 6 months) | C | (D within 6 months) | E)</td><td>(A & (B (within 6 months) | C | (D within 6 months) | E)) & F (E and F in combination)</td></tr>
</table>

<table>
<tr><th>ID</th><th>Description</th><th>A
ATC
Antipsychotics</th><th>B
ATC
Phenothiazines</th><th>Risk</th><th>Code</th></tr>
<tr><td>STOPP D12</td><td>Stop phenothiazines as first-line treatment, since safer and more efficacious alternatives exist (phenothiazines are sedative, have significant anti-muscarinic toxicity in older people, with the exception of prochlorperazine for nausea/vomiting/vertigo, chlorpromazine for relief of persistent hiccoughs and levomepromazine as an anti-emetic in palliative care).</td><td>N05AE05, N05AH04, N05AL05, N05AX08, N05AX12, N05AX13</td><td>N05AA*, N05AB*, N05AC*, N05AX07</td><td>!(A within 3 years)</td><td>!(A within 3 years) & B</td></tr>
</table>

<table>
<tr><th>ID</th><th>Description</th><th>A
ATC
Adrenergic inhalants, glucocorticoid inhalants, or anticholinergic inhalants</th><th>B
ATC
Theophylline</th><th>Risk</th><th>Code</th></tr>
</table>

**STOPP G1**	Stop theophylline as monotherapy for COPD (safer, more effective alternative; risk of adverse effects due to narrow therapeutic index).	R03A*, R03BA*, R03BB*	R03DA04	!A	!A & B

**ID**	**Description**	**A**<br>ICD-10<br>*Narrow angle glaucoma*	**B**<br>ICD-10<br>*Prostatism or urinary retention*	**C**<br>ATC<br>*Antimuscarinic bronchodilators*	**Risk**	**Code**
**STOPP G3**	Stop antimuscarinic bronchodilators (e.g. ipratropium, tiotropium) with a history of narrow angle glaucoma (may exacerbate glaucoma) or bladder outflow obstruction (may cause urinary retention).	H40.2	N40*, R33*	R01AX03 , R03AL*, R03BB*	A | (B within 6 months)	(A | (B within 6 months)) & C

**ID**	**Description**	**A**<br>ATC<br>*Nonsteroidal anti-inflammatory drugs*	**Risk**	**Code**
**STOPP H6**	Stop long-term NSAID or colchicine (>3 months) for chronic treatment of gout where there is no contraindication to a xanthine-oxidase inhibitor (e.g. allopurinol, febuxostat) (xanthine-oxidase inhibitors are first choice prophylactic drugs in gout).	M01A*, M01BA*, N02BA*	All	A, if more than 90 DDDs within 6 months

**ID**	**Description**	**A**<br>ICD-10<br>*Coronary, cerebral or peripheral vascular disease*	**B**<br>Procedure codes<br>*Coronary implants (CABG or PCI)*	**C**<br>ATC<br>*COX 2 selective nonsteroidal anti-inflammatory drugs*	**Risk**	**Code**
**STOPP H7**	Stop COX-2 selective NSAIDs with concurrent cardiovascular disease (increased risk of myocardial infarction and stroke)	I20*, I21*, I22*, I23*, I24*, I25*, E10.5, E11.5, E14.5, I70.2, I73.9, I74.3, I63*, I64*, I65*, I66*, G45*	KFN*, KZFX01	M01AH*	A | B	(A | B) & C

**ID**	**Description**	**A**<br>ICD-10<br>*Dementia*	**B**<br>ICD-10<br>*Delirium*	**C**<br>ICD-10<br>*Narrow angle glaucoma*	**D**<br>ICD-10<br>*Prostatism and urinary retention*	**E**<br>ATC<br>*Bladder antimuscarinic agents*	**Risk**	**Code**
**STOPP I1**	Stop antimuscarinic drugs with dementia, or chronic cognitive impairment (risk of increased confusion, agitation) or narrow-angle glaucoma (risk of acute exacerbation of glaucoma), or chronic prostatism (risk of urinary retention).	F00*, F01*, F02*, F03*, G30*, G31.0, G31.1, G31.8,	F05*	H40.2	N40*, R33*	G04BD01, G04BD02, G04BD03, G04BD04, G04BD05, G04BD06, G04BD07, G04BD08, G04BD09, G04BD10, G04BD11	A | (B within 6 months) | C | (D within 6 months)	[A | (B within 6 months) | C | (D within 6 months)] & E

**ID**	**Description**	**A**<br>ICD-10<br>*Heart failure*	**B**<br>ATC<br>*Thiazolidenediones (e.g. rosiglitazone, pioglitazone) or combinations*	**Risk**	**Code**
**STOPP J2**	Stop thiazolidenediones (e.g. rosiglitazone, pioglitazone) in patients with heart failure (risk of exacerbation of heart failure)	I50*, I11.0, I13.0, I13.2	A10BD03, A10BD04, A10BD05, A10BD06, A10BD09, A10BD12, A10BG*	A	A & B

**ID**	**Description**	**A**<br>ICD-10<br>*History of breast cancer*	**B**<br>ICD-10<br>*History of venous thromboembolisms*	**C**<br>ATC<br>*Estrogens (or combinations)*	**Risk**	**Code**	**Note**
**STOPP J4**	Stop oestrogens with a history of breast cancer or venous thromboembolism (increased risk of recurrence).	C50*, D05*	I80*, I81*, I82*, I26*	G02BB*, G03AA*, G03AB*, G03CA01, G03CA06, G03CA07, G03CA09, G03CA53, G03CA57, G03CB*, G03CC*, G03EA*, G03EB*, G03F*, G03HB*, G03XC*, **G03CA03, G03CA04**	Female sex & (A | B)	(Female sex & (A | B)) & C	Estradiol (ATC: G03CA03) only, if whoadmvej = "DEPPLA"|"GEL"|"TABFILM", and Estriol (ATC: G03CA04) only, if if whoadmvej = "TAB"|"VAG"

**ID**	**Description**	**A**<br>ICD-10<br>*Hypogonadism*	**B**<br>ATC<br>*Androgens*	**Risk**	**Code**

**STOPP J6**	Stop androgens (male sex hormones) in the absence of primary or secondary hypogonadism (risk of androgen toxicity; no proven benefit outside of the hypogonadism indication).	E29.1, E89.5, Q98.0, Q98.1, Q98.2, Q98.4	G03B*	Male sex & !A	(Male sex & !A) & B

**ID**	**Description**	**A**<br>ICD-10<br>*Ortostatic hypotension or syncope*	**B**<br>ATC<br>*Vasodilator drugs (alpha-1 blockers, calcium channel blockers, angiotensin-converting-enzyme inhibitors, or angiotensin II receptor blockers)*	**Risk**	**Code**
**STOPP K3**	Stop vasodilator drugs (e.g. alpha-1 receptor blockers, calcium channel blockers, long-acting nitrates, ACE inhibitors, angiotensin I receptor blockers,) with persistent postural hypotension i.e. recurrent drop in systolic blood pressure ≥ 20mmHg (risk of syncope, falls)	I95.1, R55*	C01DA*, C02CA*, C02LE01, C07AG*, C07BG*, C07CG*, C08*, C09*, G04CA*	(A within 6 months)	(A within 6 months) & B

**ID**	**Description**	**A**<br>ATC<br>Selective beta-2-adrenoreceptor agonists	**B**<br>ATC<br>Adrenergics in combination with anticholinergics incl. triple combinations with corticosteroids	**C**<br>ATC<br>*Anticholinergic agents*	**D**<br>ATC<br>Antihistanines for systemic use	**E**<br>ATC<br>Carboxamide derivatives (antiepileptics)	**F**<br>ATC<br>Antipsychotics	**G**<br>Anxiolytics	**H**<br>ATC<br>Drugs for urinary frequency and incontinence	**I**<br>ATC<br>Non-selective monoamine reuptake inhibitors (antidepressants)	**Risk**	**Code**
**STOPP M**	Stop concomitant use of two or more drugs with antimuscarinic/anticholinergic properties	R03AC06	R03AL01, R03AL02, R03AL03, R03AL05, R03AL07, R03AL09	R03AX03, R03BB01, R03BB04, R03BB05, R03BB06, R03BB07	R06AA*, R06AB*, R06AC*, R06AD*, R06AE01, R06AE03, R06AE04, R06AE05, R06AE06, R06AE51, R06AE53, R06AE55, R06AX01, R06AX02, R06AX03, R06AX04, R06AX05, R06AX08, R06AX09, R06AX13, R06AX15, R06AX16, R06AX17, R06AX23, R06AX27, R06AX53, R06AX58	N03AF01, N03AF02	N05AA01, N05AA02, N05AA04, N05AB02, N05AB03, N05AB04, N05AC01, N05AC02, N05AF04, N05AG02, N05AH02, N05AH03	N05BB01, N05BB51	G04BD01, G04BD02, G04BD03, G04BD04, G04BD05, G04BD06, G04BD07, G04BD08, G04BD09, G04BD10, G04BD11	N06AA*	All	Two or more drugs from each of the subgroups concurrently (in combination)
